# Building a model of navigational strategies for queer undergraduate students in STEM

**DOI:** 10.3389/fsoc.2023.1293917

**Published:** 2023-11-30

**Authors:** Matthew Voigt, Margaret Ann Bolick, Destinee Cooper, Sarah Otterbeck, Abigail Rose Smith, Chloe Wright, Clara Holloman

**Affiliations:** Department of Engineering and Science Education, Clemson University, Clemson, SC, United States

**Keywords:** queer, STEM, LGBTQQIP2SA+, discourses, navigational strategies, trauma, cognitive

## Abstract

**Introduction:**

There is a critical need to foster inclusive educational spaces for Queer identifying students and to resist oppressive structures that seek to marginalize and inflict trauma on students because of their gender or sexual identity.

**Methods:**

Drawing on thematic analysis and Queer theory, we interviewed 11 Queer identifying STEM students to understand the navigational strategies they leveraged within higher education environments related to their Queer identity.

**Results:**

We developed a cyclical model of navigational strategies employed by Queer STEM students that involved evaluating the environments, performing psychological identity calculations, and engaging in behavioral actions. Students evaluated the environment by attending to the diversity of gender representation, presence of other Queer individuals, and contextual factors conveyed based on disciplinary expectations. Students engaged in psychological identity calculations whereby they assessed beliefs about the relevance, importance, and fears related to their Queer identity, with few perceiving any benefits. Behavioral actions resulted in students building a chosen community, disclosing or shelving their queer identity, and advocating for representation.

**Discussion:**

In order to support Queer students to thrive in educational contexts, researchers and practitioners should examine ways to increase representation, use inclusive pedagogical strategies, and understand the relevance of Queerness within disciplinary fields. Questioning the relevance or presence of Queerness in higher education environments only further serves to oppress, inflict trauma, and marginalize Queer students.

## 1 Introduction

Representation matters! And thus, it is important that we build educational spaces so that individuals feel comfortable “coming out” and do not fear harm or potential trauma from others. Coming out in educational spaces is often impacted by situational variables that relate to the climate and reported comfort levels in disclosing sexual identity. Educational research has suggested that Queer students are more comfortable coming out in classes where they know the other students (Eliason and Turalba, 2019), where they perceive the classroom climate and instructor as more accepting (Lopez and Chims, 1993), and are influenced by the specific disciplinary environment (Bilimoria and Stewart, 2009; Leyva et al., 2016; Yoder and Mattheis, 2016). As such, there is a need to better understand the nuanced factors impacting Queer students’ experiences that are situated within disciplinary contexts and instructional environments. This study aims to explore the experiences of Queer students who are majoring in STEM (Science, Technology, Engineering, and Mathematics) fields and the navigational strategies they employ to be successful within the spaces of higher education. This work is imperative for increasing the participation, success, and retention of Queer students.

There has been a push to broaden the participation of students with marginalized identities in STEM over the past several decades; however, students with a Queer identity are chronically forgotten, understudied, and underrepresented in STEM spaces. As a result, there is limited prior research on Queer students’ experiences in undergraduate STEM courses (Cech and Waidzunas, 2011; Cooper and Brownell, 2016; Kersey and Voigt, 2021) and even less on Queer STEM students’ experiences in different higher education environments.

Queer students in the arts, humanities, and social sciences often describe a more welcoming and inclusive climate as compared to students in STEM fields. For example, the voices and lives of Queer individuals are often represented in the curriculum for arts and humanities, but in STEM this is often seen as “tangential” to the subject (Hughes, 2018). Based on existing literature, we know Queer students are comparatively more likely to change from a STEM to a non-STEM major than students who do not report having a Queer identity (Hughes, 2018). Additionally, Queer students have described STEM classrooms as “not being a welcoming or accepting space” (Cooper and Brownell, 2016), which is further complicated in classrooms that leverage active learning (group work, class discussions, etc.) which often “increases the relevance of students’ Queer identities due to increased interactions” with peers (Cooper and Brownell, 2016). These class structures and interactions are often viewed as unsafe spaces for students with Queer identities. Through social interactions within these climates, Queer identity can be marginalized and oppressed even without students disclosing one’s Queer identity. These marginalizing forces can occur through the presence of microaggressions (e.g., derogatory statements, invalidations, insults) that creates barriers for students in coming out (Vaccaro, 2012; Vaccaro and Koob, 2018). For instance, 99% of Queer youth report hearing the derogatory use of phrases such as ‘that’s so gay’ or ‘you are so gay’ in school (Kibirige and Tryl, 2013). These nondescript microaggressions impress that Queer identities are something to be avoided and concealed, especially in the social and educational context of school environments. The more these comments are tolerated, the more oppressive tension Queer youth feel to hide their identities as they grow older. Therefore, researchers and practitioners must consider how to make learning spaces more inclusive and equitable for Queer students.

The perceived objectivity within STEM creates dissonance among students that their Queer identity is irrelevant and should not impact their experiences. For instance, although Queer students report neutral climates in STEM spaces, this is due in part because they report not having connections with Queer communities and do not believe their Queer identity relates to the discipline (Gunckel, 2009; Fischer 2013; Cech, 2015; Hughes, 2018; Haverkamp et al., 2021). These students often cast STEM as an escape from their Queer identity since “STEM creates objective viewpoints where orientation is not considered…[and] gender and sexuality are not important to the efficiency of work” (Smith, 2014, p. 60). This belief that Queer identity is irrelevant results in Queer students feeling uncomfortable revealing their sexual orientation in STEM spaces, because of their desire to not make others uncomfortable, and since coming out creates a sense of constant vulnerability that means staying closeted is safer and easier in these spaces (Smith, 2014). When you combine students’ personal views of coming out with pressure within the field to depoliticize STEM and thus remove any mention of social identities, it results in the erasure and oppression of Queer identities in STEM (Cech, 2015). In order to advance the culture of equity and inclusion for Queer students in STEM, there may be benefits to studying their experiences in non-STEM learning spaces as well.

As such, our goal is to better understand the experiences of Queer STEM students in higher education by investigating the following research question: *What are the navigational strategies that Queer STEM students leverage to support their sense of safety, belonging, and ability to thrive in higher education?*

## 2 Literature review

### 2.1 Previous related research queer in STEM

Despite efforts to increase representation of racial, ethnic, and sexual minority groups in STEM, a recent national longitudinal survey confirms that Queer students are 7% less likely to be retained in STEM fields than their heterosexual peers in postsecondary education (Hughes, 2018). This study also found that both gender and sexual minority status were negative predictors of STEM retention in their multilevel regression model. Hughes (2018) noted that faculty and administrators may attribute the differences in retention to academic preparation and participation in experiences known to support success in STEM (such as undergraduate research), but the difference in retention was still observed when controlling for these factors. In other words, Queer students “who are as academically prepared, or even more prepared than, their peers leave STEM at higher rates” (Hughes, 2018, pg. 3).

One exploratory study by Cooper and Brownell (2016), specifically considered the experiences of Queer students in an undergraduate biology lecture course that incorporated collaborative learning groups and other active-learning strategies. They found that Queer biology students do not perceive biology classrooms to be welcoming to their social identities, but engagement in active-learning increased the relevance of their social identities due to more opportunities for interaction with peers (Cooper and Brownell, 2016). Cooper and Brownell (2016) also determined that collaborative group work may increase the comfort level of Queer students who may get to collaborate with students who share their identity or are accepting of it, but may also lead to discomfort when grouped with students who are not perceived as accepting of their social identity. They reported that navigating these group dynamics may lead to an increased cognitive load for Queer students who are working through academic content while also grappling with fears of being stigmatized, misgendered, or unaccepted by their peers (Cooper and Brownell, 2016). Queer students are very mindful of who they are working with and desire the autonomy to choose who they work with to ensure their safety and sense of belonging (Cooper and Brownell, 2016). Similar to Hughes, this study is a call to action for faculty and researchers to better understand and improve the climate of STEM learning spaces for Queer students to ultimately increase their retention in the field.

There is an increase in literature surrounding Queer postsecondary STEM students and how they perceive STEM fields in conjunction with their Queer identities. In two recent studies, participants described STEM fields as devoid of identity and objective; yet, additionally cited that their Queer identities felt excluded within STEM fields (Kersey and Voigt, 2021). More intricately, the studies found that postsecondary trans women in STEM described gender-based biases within their STEM courses referencing the overt oppression participants would face when presenting as female versus when presenting as male which not only confirms the presence of gender-based bias but transfers to participants feeling as though their Queer identities are unwelcome within STEM spaces (Kersey and Voigt, 2021). The majority of participants continued to allude to STEM spaces feeling exclusionary to their Queer identities even when viewing their own Queer identities as strengths (Kersey and Voigt, 2021).

In order to increase participation of women and Queer individuals within STEM fields, it is necessary to question the gendered nature of STEM fields through the lens of Faulkner’s technical/social dualism (Leyva et al., 2016). Leyva and colleagues found that it is impossible to capture the unique experiences women and Queer individuals have within engineering spaces as a collective whole; instead, there should be an emphasis on capturing individual experiences within engineering to develop a more nuanced understanding of how marginalized student groups negotiate their identities within these masculinized spaces (Leyva et al., 2016). Utilizing methodologies that focus on capturing all aspects of an individual’s story (e.g., ethnography) has the potential to help document the reality of Queer individuals’ experiences within engineering (Leyva et al., 2016).

The recommendations emerging from the literature clearly point to a need for pedagogical changes to increase inclusivity within STEM classrooms. Faculty must increase their awareness and understanding of Queer identities and issues, known as allied competencies (Jones et al., 2014), to support their ability to foster a safe and inclusive classroom community for Queer students (Cooper and Brownell, 2016; Hughes, 2018). The major component of allied competencies needed in this work have been operationalized and measured (Jones et al., 2014; Lopez-Saez et al., 2020; López-Sáez et al., 2022) to include *knowledge and awareness* about the experiences of queer people, *openness and support* of collective action, and *oppression awareness* by understanding privilege and the daily injustices that shape Queer life experiences. These competencies can be developed and refined through participation in Safe Zone workshops and active involvement in organizations like oSTEM (Out in STEM; Cooper and Brownell, 2016; Kersey and Voigt, 2021). Queer identity affirming teaching practices that have the potential to provide an inclusionary space for Queer postsecondary STEM students include providing space for students to share their identity and pronouns, incorporating examples of Queer scientists in the curriculum, and challenging gender-based biases (Leyva et al., 2016; Kersey and Voigt, 2021).

### 2.2 Queer theory

Queer theory was adopted for our study because of its focus on achieving equity for Queer individuals across a variety of contexts (McWilliams and Penuel, 2017). Queer theory aims to disrupt binary assumptions of gender and sexuality by questioning processes that “define and categorize people, ideas, identities, and institutions” (Gunckel, 2009, p. 63). Similar to Kersey and Voigt, we avoided “placing boundaries around categories like *STEM* and *Queer* and opted to let our participants decide if these words applied to them or not” (2021, p.736). We also disrupt binary assumptions of gender and sexuality to consider whose perspectives may be missing in learning space and how those views might impact understanding and outcomes (Waid, 2023).

It was important that we situate our work within the framing of Queer theory because we do not want to focus simply on the acceptance of those in the Queer community by the cisgender, heterosexist majority, but rather on questioning discourses that “position Queer identity as excluded and irrelevant to the pursuit of STEM” (Voigt, 2020, p. 266). The questioning stance that Queer theory advocates for allows us to more effectively address the systemic issues that lead to chilly and overtly hostile climates for Queer students in STEM learning spaces (Hughes, 2018). By investigating how Queer STEM students navigate these less accepting spaces, we intend to pinpoint what specific issues span different spaces within higher education and focus on the assets Queer students bring to these spaces. Applying Queer theory to education significantly benefits everyone, not just those who identify as Queer (Gunckel, 2009). It opens new knowledge and ways of understanding that may centralize, but also extends beyond the lives and experiences of those that identify as Queer (Gunckel, 2009; McWilliams and Penuel, 2017).

## 3 Methods

### 3.1 Study design

**Figure 1:**
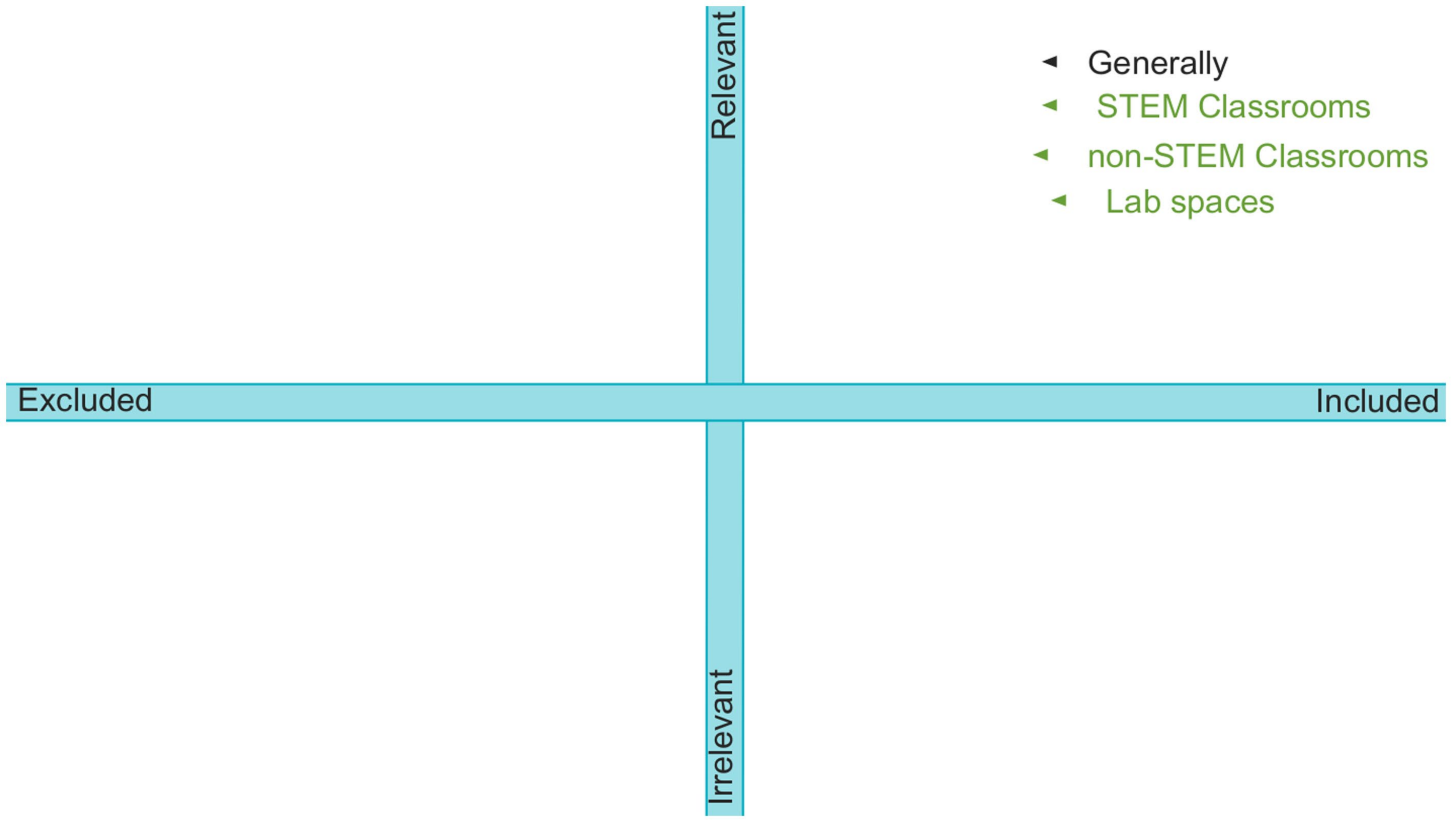
Participants placed how relevant/irrelevant their queer identity felt and how included/excluded they felt in four spaces: generally, STEM classrooms, non-STEM classrooms, and lab spaces.

This study is intended to elevate and advocate for the voices of Queer STEM individuals within the realm of higher education. The emphasis on sharing and amplifying these voices align with the transformative paradigm, because it utilizes an asset-based lens when focusing on populations that experience discrimination and oppression from dominant cultural forces, in this case those students who have a Queer spectrum identity (Mertens, 2010). The findings from this study and a “call to action” were presented to the Hagler University LGBTQ commission as a mechanism for transformative change. In addition, we acknowledge the ways in which our research design does not fully align with the transformative paradigm given the limited relationship development with our participants; however, members of the Queer community were a part of the development, implementation, and analysis via the research team composition (see Section 3.4 Positionality). We leveraged a mixed-methods design approach drawing primarily from semi-structured qualitative interviews but also included a quantitative component referred to as the exclusion-irrelevancy plane (see Figure 1) whereby students placed and described the relevancy/irrelevancy and inclusion/exclusion toward their Queer identity within different spaces in higher education (Voigt, 2020). Therefore, the design of the study is classified as mixed methods as it draws on the collection, analysis, and blending of qualitative and quantitative data (Creswell et al., 2011). More specifically, the mixed methods design can be classified as a complementarity mixed methods design as this study works to provide a more holistic understanding of Queer STEM students in higher education (Creamer, 2017).

### 3.2 Participants and context of interviews

Students were recruited from an R1, predominantly white institution (PWI) located in South Carolina within the United States, referred to as Hagler^1^ University. We used the following inclusion criteria for participant recruitment and selection: currently enrolled during the Spring 2022 semester at Hagler University, self-identified as Queer, and pursuing a STEM degree. Snowball sampling and university list-servs were used to recruit participants, resulting in 11 participants in total. A summary of participants including their pseudonym, pronouns, major, sexuality, gender, and a self-description (using the question “what three words would you use to describe yourself?”) is included in Table 1.

**Table 1:** Description of participants self-described identities.

#	Pseudonym	Pronouns	Major	Sexuality	Gender	Self-description
1	Chase	they/them	Plant & Environmental Sciences	Pansexual	Non-binary or Genderfluid	“Under the trans umbrella”
2	Horseshoe Crab	she/her	Mathematical Science	Lesbian	Non-binary or Genderfluid	Bitch, Sympathetic, Lesbian
3	Sheldon	he/him	Electrical Engineering	Gay	Cis-man	Black, Friendly, Nerdy, Logical
4	Blaise	they/them	Psychology	Gay	Non-binary or Genderfluid	Me, Open, Upstanding
5	Chris	they/them	Forestry Resource Management	Queer	Agender	Loyal, Inquisitive, Passionate
6	Cass	she/her	Mechanical Engineering	Lesbian	Cis-woman	Loyal, Fun, Hard Working
7	Sam	she/they	Architecture	Questioning (Asexual, Lesbian, or Pansexual)	Cis-woman, Non-binary or Genderfluid	Anxious, Different, Open-Minded
8	Niko	they/them	Conservation Biology	Aromantic	Trans-man, Non-binary or Genderfluid	Inquisitive, Kind, Queer
9	Alex	she/her	Mathematical Science	Bisexual	Cis-woman	Independent, Determined, Active
10	Emma	she/her	Computer Science	Lesbian	Cis-woman	Blunt, Pragmatic, Layered (like an onion not a parfait)
11	Phin	he/him	Animal & Veterinary Science	Gay	Cis-man	Joyful, Crazy, Weird, Passionate

The 11 semi-structured interviews took place over Zoom to adhere to COVID-19 safety protocols as well as to easily record participant affect and download a transcript from each interview. The interview incorporated background questions on participants’ “coming out” journeys, experiences within different spaces at their current institution, and how they perceive their Queer identities within these spaces (see Appendix A for protocol). Additionally, the interview included three interactive tasks using Google JamBoards for students to talk through their reasoning as they positioned how they perceived their Queer identity in different environments in higher education on a one-dimensional scale of included/excluded, a one-dimensional scale of relevancy/irrelevancy, and a two-dimensional excluded/irrelevancy space (Figure 1). Each interview lasted between 37 min and 1 h and every participant responded to the question using the two-dimensional excluded/irrelevancy space.

To attend to differences and acknowledge the lived experiences of our participants, quotes will be used to explain the salient points of each theme. Participants are also addressed using the pseudonyms and pronouns that they selected in Table 1.

### 3.3 Data analysis

**Figure 2:**
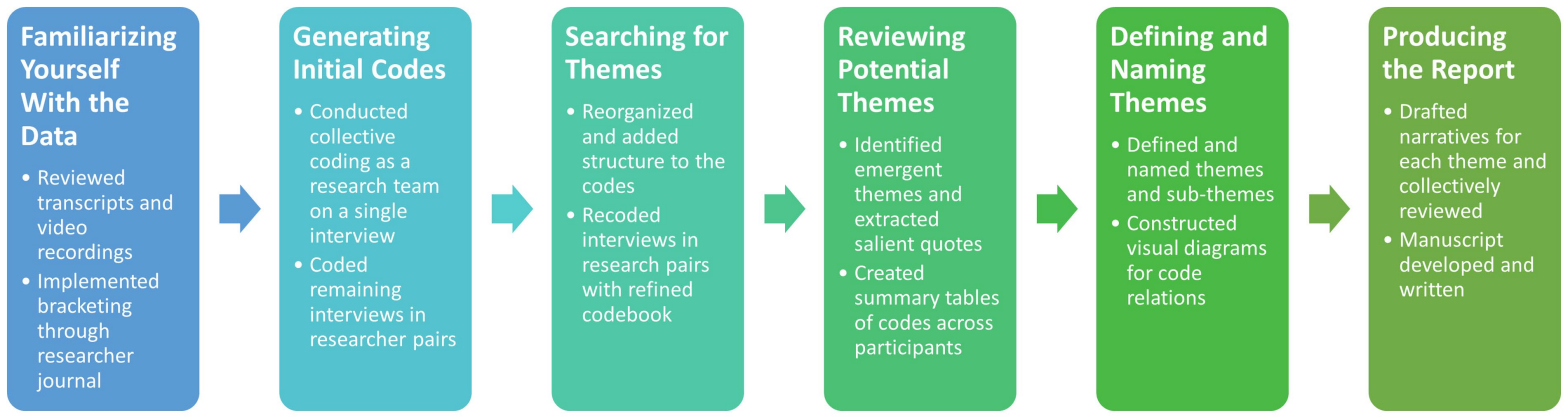
Visual depiction and summary of the six stages of thematic analysis that guided the research study.

Thematic analysis was used as the primary qualitative data analysis method because of its accessibility and ease of use when formulating patterns across multiple data sources (Alhojailan, 2012; Braun and Clarke, 2012). A robust data analysis plan was developed (Green et al., 2007; Braun and Clarke, 2012) that incorporated the four major characteristics of a high-quality paper: immersion in the data, thoroughly coding the data, developing categories from the produced codes, and creating themes that accurately reflect the data. These four characteristics provided depth to the thematic analysis process and were divided into six different phases: (1) Familiarizing Yourself With the Data, (2) Generating Initial Codes, (3) Searching for Themes, (4) Reviewing Potential Themes, (5) Defining and Naming Themes, and (6) Producing the Report (Braun and Clarke, 2012). A description of each phase is included in the sections below and is visually depicted in Figure 2.

#### 3.3.1 Familiarizing yourself with the data

After interviews were recorded and transcribed, the entire research team initially reviewed one transcript (Horseshoe Crab) separately. The remaining 10 interviews were divided among the research team in pairs of undergraduate and graduate students. Two of the three pairs included members of the Queer community to engage in member checking. Almost every pair contained someone who conducted an interview providing each team with more insight into the data, an important piece of the research team immersing themselves in data (Green et al., 2007). Every pair familiarized themselves with the interviews by rewatching the recordings, reading the transcripts multiple times, and actively taking notes on pieces of information that stood out in the individual interview. By actively reading through transcripts and drawing out pieces of important large-scale information, the team had references to revisit when coding the data (Braun and Clarke, 2012). Additionally, during the familiarization stage, the team bracketed in a research journal any personal reactions they had to what they were listening to and reading the transcripts.

#### 3.3.2 Generating initial codes

Throughout the coding process, the research team utilized MAXQDA software as a convenient way to import, code, and share data across the team. Initially, all members of the research team individually inductively coded one interview (Horseshoe Crab). The codebooks from the one interview were merged, reconciled, and redistributed as a guiding codebook for the rest of the interviews. All of the interviews were coded by referencing the initial codebook and adding codes specific to new units of meaning in new interviews. Once every interview was coded, the new codebooks were merged to create a codebook that captured the whole data set.

#### 3.3.3 Searching for themes

The codebook was reorganized into clusters of codes relaying similar, the same, or related information. Codes that were the same or had similar units of meaning were collapsed into a singular code by using constant comparison between transcripts (Ryan and Bernard, 2003). Codes that were related were categorized under larger headings. These categories and clusters of codes within each category provided insight into the frequency with which the codes were being used. Frequency is a valuable initial determinant of a theme but was cross-referenced with the research question to make sure the theme was relevant (Ryan and Bernard, 2003; Vaismoradi et al., 2016). Based on the frequency of each category and the codes within each category, themes started to emerge from the data. After developing categories in the codebook, the coded transcripts were recoded to incorporate the newly robust codebook. To prevent bias around emerging themes, the research team did not code blank transcripts to prevent preconceived theme development. By recoding the transcripts with additional categorical codes, emerging themes became more evident across the data set.

#### 3.3.4 Reviewing potential themes

Once each transcript was recoded with additional categorical codes, quotes were pulled out from each participant’s transcript and sorted under each emerging theme. The collection of quotes for each participant was summarized under each emerging theme to fully capture how the participant connected to the theme. Tables were created for each emerging theme and each participant had their own column to help visualize themes across all participants. By using the table, it was easier to visualize how participants connected to the emerging themes and how each emerging theme connected to the other. During the review process, we identified varying interpretations of individual themes and how they connected, collapsed themes that did not have enough data to support them, and reworded themes to better encapsulate the data (Braun and Clarke, 2012).

#### 3.3.5 Defining and naming themes

Themes were summarized to ensure there was enough depth and data to support the findings. Data that was already selected in Phase 4 was reviewed and quotes that best demonstrated the theme were selected to illustrate the collective narrative of participant voice. Analysis of the findings weaved together participant narratives and focused on analyzing the data with a Queer Theory lens. Each potential theme was connected to tangential themes and some themes turned into sub-themes under more robust themes. Themes were rewritten with the intent to tell a story that captured every participant’s experience and connection to the research questions (Braun and Clarke, 2012).

#### 3.3.6 Producing the report

The report was written simultaneously as the thematic analysis process was taking place and consistently edited depending on the new findings throughout the thematic analysis process.

### 3.4 Positionality

MV identifies as a white cisgender gay or Queer man with disciplinary backgrounds in Mathematics and Math Education. MAB identifies as a white heterosexual cisgender woman with disciplinary backgrounds in Biomedical Engineering and Math Education. DC identifies as a Black cisgender heterosexual woman with disciplinary backgrounds in Chemistry and Chemistry Education. SO identifies as a white heterosexual cis woman with disciplinary backgrounds in Mathematics and Math Education. CW identifies as a white cisgender heterosexual woman with disciplinary backgrounds in Mechanical Engineering and Spanish Language Studies. ARS identifies as a white and Native American cisgender bisexual woman with disciplinary backgrounds in Genetics, Biochemistry, and Psychology. CH identifies as a white non-binary lesbian (or woman-adjacent) with an academic background in Biochemistry. CH is currently pursuing an M.D. with an intended career in obstetrics and gynecology.

Understanding our positionalities and making these apparent throughout the research process supports the validity and reliability of our findings. This was enacted by having the Queer identified researchers develop the interview protocol to centralize the questions and experiences of Queer individuals. Queer identified researchers also conducted the interviews with Queer student participants to support comfort and understanding during the data collection. During the coding and analysis, where possible, we paired Queer identified researchers with non-Queer identified researchers to code and reconcile the data to support communicative validity of our code book. During the writing phase, all of the researchers contributed and reviewed the manuscript to ensure it captured our beliefs and framing of the research findings.

## 4 Results

**Figure 3:**
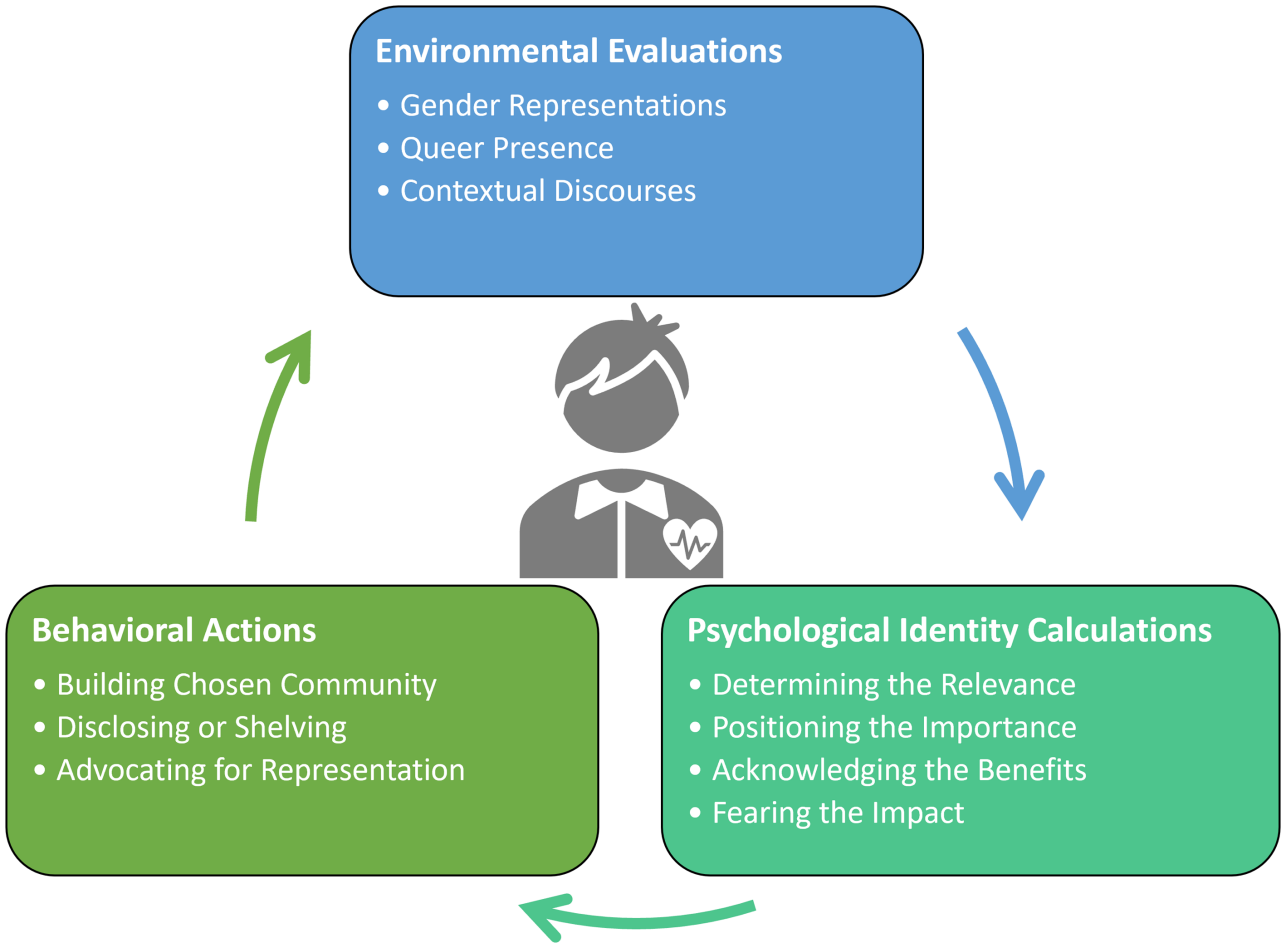
Visual model of Queer STEM students navigational strategies in higher education environments.

There were three main mechanisms or navigational strategies that Queer STEM students in this study leveraged to support their sense of safety and belonging in higher education: *Environmental evaluations*, *Psychological identity calculations*, and *Behavioral actions*. Environmental evaluations encompassed strategies to read the environments for Queer safety. Psychological identity calculations encompassed the internal cognitive processes that students described they would execute before taking an action. Behavioral actions encompassed the strategies and overt acts that students used to navigate higher education spaces. Each of these themes and their sub-themes is discussed in greater detail in the following sections and visually presented in Figure 3. We note at the onset of our results that few students conveyed a sense of thriving in higher education, most discussing personal safety or belonging, which may be an indication that the environment has not yet satisfied the basic safety needs for Queer students in higher education.

### 4.1 Environmental evaluations

Participants in this study were keenly aware and adept at performing assessments and evaluations of their local environment to attend to factors that might indicate the level of inclusion and safety of their Queer identity within the space. These evaluations were performed constantly in the moment, were often re-assessed with additional context, and built upon larger societal and historical discourses about Queerness in the United States and within STEM. Three major sub-themes that guided students evaluations were: *Gender representation, Queer presence*, and *Contextual discourses*.

#### 4.1.1 Gender representation

Eight of the participants in the study discussed how gender representation, and specifically the presence of cisgender white men in spaces, was an indicator of less Queer inclusion. Furthermore, it was not only the gender representation of the environment, but how gender interplayed and intersected with issues of race and the participants sexual identity. Participants described attending to the environmental factor of gender representation often using various vocabulary such as “STEM is very straight cis male dominated…boys club,” “white male people…dominating in that class,” “computer science…is like very, very male dominated…toxically straight,” “dominated by white men,” “straight men,” “my major is very like classic cishet white generally um country boys.” It is worth noting how much of the language included the terminology of “dominating,” a descriptor that conveys power, and subjugation of Queer people. Students were evaluating the environment for the representation of gender diversity as an indicator of their safety. Chase (Pansexual Non-binary) captured this sentiment saying, “it just feels sort of scary because I do not want to get targeted by someone for being different and Queer.”

Queer women especially noted how the intersection of gender and sexual identity impacted the way they understood the environment. For instance, Cass (Lesbian Cis-woman) discussed if you told a straight man your sexual identity they would “assume they can talk about women in a certain way…misogynistic and stuff” which can result in issues developing genuine connections with other students. In a similar vein, Horseshoe Crab (Lesbian Non-binary) mentioned how straight men can be exclusionary to Queer women who do not have a sexual interest in them:

*there’s a lot of men in there that normally do not geehaw*^2^ *with Queer women or just women that don’t show a lot of interest in them, and they are more dominating in that classroom…lean towards the superiority complex and don’t really seem open to allowing Queers into their spaces*.

Emma (Lesbian Cis-woman) described this type of environment as “toxically straight,” often because many guys have “never spoken to a girl before or at least they act like it” and thus any sort of interaction with them appears that you are “giving him the time of the day.” Emma said this can be “difficult to navigate” as they do not want to out themselves but they do not have another reason to provide why they are not sexually/romantically interested in them.

However, we should caution that it is not enough to address toxic masculinity and increase the representation of cisgender women in STEM to improve the environment for Queer students, we must move beyond the gender binary. As an example, Chase (Pansexual Non-binary) discussed how their automatic invitation and inclusion in a women in engineering program while helpful did not feel inclusive:

*Especially the people that I know that don’t identify as female but are in the [women in engineering] email list or [program] I know that for them that’s really hard… I was assigned a [women in engineering] mentor and it did feel weird to me because I don’t identify as a woman. And that’s something that my freshman engineering professor said, he was like we need more women in engineering, and I was just going with it, because it’s specifically for women and so they just take everyone who was born female at birth, who you know, has that gender marker and if they’re in engineering they will automatically put you in [program] with a mentor and I can’t change how people perceive*.

#### 4.1.2 Queer presence

All of the participants in the study discussed how they evaluated the environment by attending to the presence of other Queer individuals (or Queer-inclusive allies) within the space; yet, this was not just a passive action of attending to Queer visibility but was often an active result of building and developing Queer-inclusive communities in the spaces around them (discussed further in the *Building Chosen Community* section). Given the sometimes less visible nature of Queer identity, determining Queer presence often necessitated active strategies to assess the Queerness or inclusiveness of others present in the environment. Queer students evaluated the presence of Queer individuals usually through social media, friend groups developed outside of academic spaces, or through connections with roommates.

Students used social media (TikTok, Tinder, and Instagram) to identify individuals within their physical proximity that they used to determine Queer presence. For instance, Cass (Lesbian Cis-woman) described how her friend created a group chat with all of the Queer women that she knew from Tinder. Another student discussed how seeing pronouns on a peers’ Instagram page was a sign of inclusion that put them at ease. Given that Queer undergraduate students are mostly digital natives, the ways in which social media and technology can be leveraged to proactively foster these types of environments is an area of needed research.

Seven of the students specifically discussed the nature of finding other Queer individuals in higher education resulting in an “instant bond,” and mutual understanding. Chase (Pansexual Non-binary) described this connection in the following way:

*instantly connect with people in your community, if you find another Queer person in your major or in STEM or in your dorm … you normally have an instant connection, because you know you’re a part of that same community and that always that can lead to you know a friendship, a lot quicker than it would like if I had met another random person who wasn’t in the Queer community*

Cass (Lesbian Cis-woman) described this connection in a similar way, where you have mutual understanding of the struggles of being Queer and coming out, which results in “instantly getting each other” and when this happens within your major there is an even deeper level of understanding.

It is worth noting that an instant connection with someone who shares a marginalized identity is not unique to Queer individuals, the same has been documented with race (Jackson and Hui, 2017), gender (Lane et al., 2023), language (Calleros and Zahner, 2023), and other marginalized identities (Boyd, 2023). However, what is unique is that Queer individuals have often found community and safety with others through sexual intimacy or acknowledgment of their sexual desires. Hence why normative expectations or discourses that degenerate openness and discussions of sexuality (Voigt, 2020) are a systemic barrier to the inclusion of Queer students in STEM.

Sam (Questioning Cis-woman Non-binary), Blaise (Gay Non-binary), Sheldon (Gay Cis-man), and Chris (Queer Agender) all discussed how their roommates or living situation were a source of inclusion or indicator of Queer presence. Sam (Questioning Cis-woman Non-binary)shared:

*I’m not going to compromise, myself in like a private space for anyone. So you know we both [roommate] became friends right off the bat. It was really great, we got really lucky because half the time we feel like we’re going to get murdered by anyone else on our floor*.

Blaise (Gay Non-binary) described their roommates as really accepting, and Sheldon (Gay Cis-man) discussed how their roommates (former high school friends) were the first they came out to and how they would hang out together. Chris (Queer Agender) discussed the more formalized support of having an LGBT living and learning community within the residence hall of the university, “seeing the community that was created for that in the first class of students we had in there, shows that this sort of relevance is going to impact people’s day-to-day as soon as they get to college.”

#### 4.1.3 Contextual discourses

**Figure 4:**
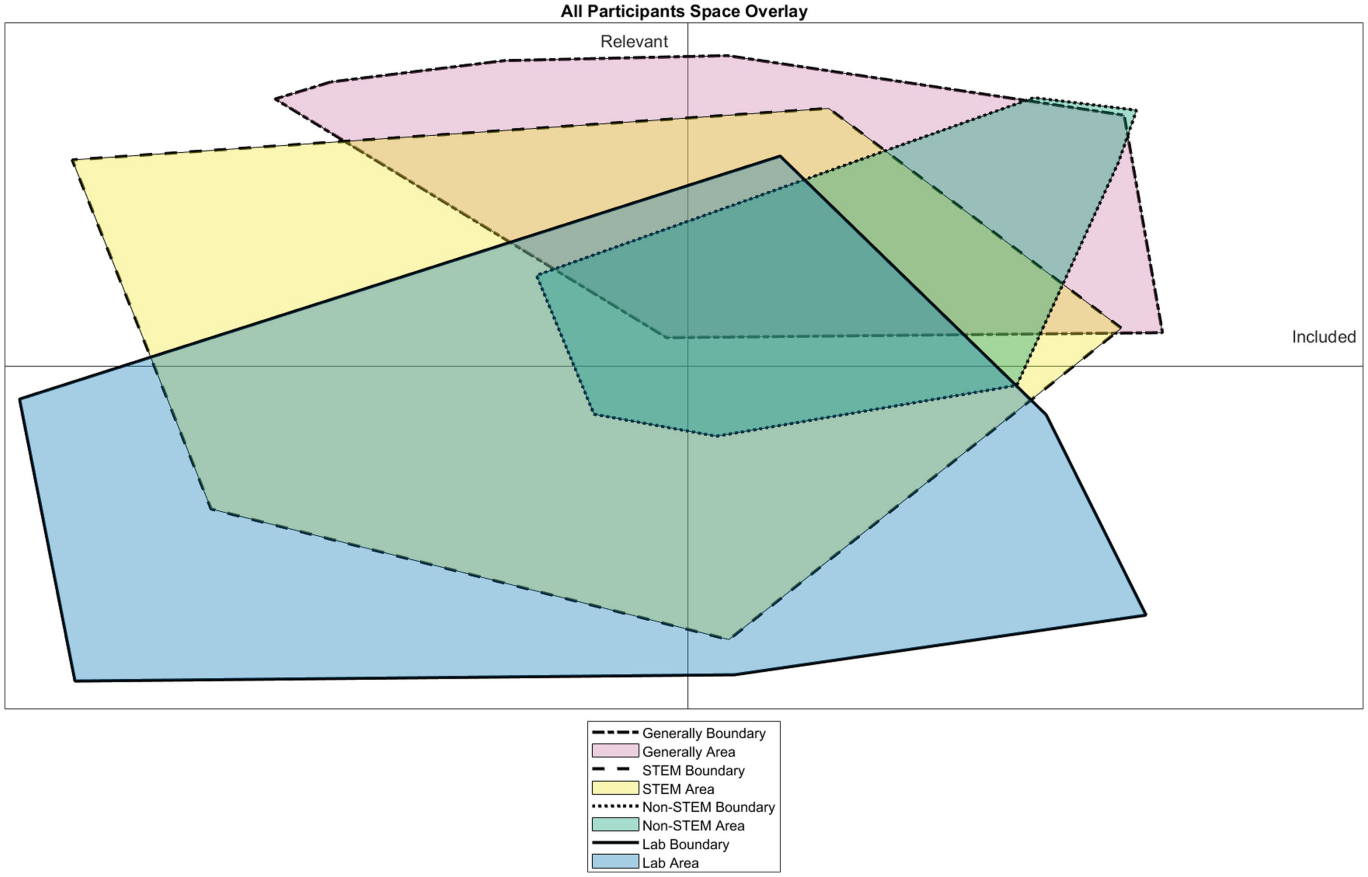
Visual depiction of the bounded area where the participants placed themselves with regards to the feelings of exclusion and irrelevance of their Queer Identity the area bounded by STEM spaces and Lab spaces primarily reside toward the exclusionary space (quadrants II and III) with Lab spaces being described as more irrelevant than any other space.

Queer students evaluated the local environment for socially constructed contextual discourses related to Queer identity and inclusion. We chose to highlight salient contextual discourses related to academic disciplines and higher education settings. Disciplinary discourses are the subtle, embedded, and normative practices within a discipline that often convey to students what is valued and important within that field. Most participants described how they evaluated STEM spaces as feeling more exclusionary than other spaces. We can see this in Figure 4, based on the location students placed themselves on the exclusion-irrelevancy space within STEM and Lab spaces being shifted more into the exclusionary plane compared to Non-STEM spaces and generally. Students described feeling more excluded in STEM because these spaces are unaccommodating to Queer identities, lack representation within the field, or the objectives and norms within STEM classrooms are not aligned with their Queer identity. Such beliefs may capture both a perception of STEM as a discipline that is aligned with cisheteronormative beliefs (Leyva et al., 2022) and learned homonegativity internalized about oneself (López-Sáez et al., 2022).

Participants described general patterns in which they felt their Queer identity was not accepted or accommodated in STEM spaces. Cass (Lesbian Cis-woman) described this feeling as, “It feels like I’m not 100% myself in these spaces. It feels like a certain mold to be seen as competent in these spaces and a lot of times unfortunately my Queerness gets pushed to the side.” Notice how Cass described being seen as competent in STEM as not aligned with also being seen as Queer. Contributing to this feeling of exclusion, students described how a lack of representation or not seeing other Queer individuals in STEM was an indicator of the exclusionary nature of those spaces. Queer representation within STEM is related to the sub-theme of Queer presence, but is more closely linked to the curriculum and professionals already in the field, as compared to peers in the same environment. One of the most frequently described indicators of Queerness being excluded in STEM was the misalignment of Queer identity with the typical norms or objectives in STEM environments that are goal-oriented and focused on technical tasks. Sam (Questioning Cis-woman Non-binary) for instance described STEM as “doing what you need to do” and is not a place to “talk with random people or work with them.” Emma (Lesbian Cis-woman) said she definitely feels excluded in STEM as a Queer person because the topic “never comes up,” “not acknowledged,” or “glossed over.” Chris (Queer Agender) described avoiding discussion of Queer topics in STEM because there is a “goal” and “we just do not talk about it [Queerness].”

A few students described moments of STEM environments feeling more inclusionary, and attributed this largely to a strong STEM identity, which fostered a sense of belonging and community. Feeling more included because of this STEM identity emerged in the interviews, with Chris (Queer Agender) discussing their love for their major, Phin (Gay Cis-man) feeling that their field was more accepting, and Niko (Aromantic Non-binary Trans-man) mentioning feeling a connection to their field. Chris (Queer Agender), Blaise (Gay Non-binary) and Horseshoe Crab (Lesbian Non-binary) described how they feel slightly more included in STEM classrooms because they knew people in their classes. Blaise also viewed science-oriented people as more open minded to the Queer community, as they actively try and understand them. As such it is worth noting that a strong STEM identity or affiliation mitigated feelings of exclusion toward the students’ Queer identity.

Participants generally expressed that non-STEM spaces feel more welcoming and inclusionary because the assignments or topics of the course were more aligned with discussions of Queer identity or they had experiences with inclusive non-STEM instructors. However, non-STEM classrooms were not viewed as a monolith of inclusion, and students had varying perspectives on which subjects were more or less inclusive. Religion, Business, History, and General Education courses were described as more exclusionary, while Art, Literature, and English were viewed as more inclusive disciplines. The majority of participants explained that non-STEM courses provided the space to reflect on their own personal identities in assignments and directly tie their identities to the material in class. Chris (Queer Agender), Horseshoe Crab (Lesbian Non-binary), Emma (Lesbian Cis-woman), Alex (Bisexual Cis-woman), Blaise (Gay Non-binary), all described non-STEM being to some degree more inclusive because Queerness is “something that is acknowledged,” there are “way more discussions, representations,” “more conversations about it,” or because they are “designed to be inclusive.” Sam (Questioning Cis-woman Non-binary) and Chris both mentioned particular experiences in non-STEM classes where they had instructors who were explicitly inclusive, used pronouns, and stated the classroom was a “safe space” where “we do not judge.” Both of them felt this was not typical of instructors and not all instructors can be “this amazing.” It is striking and somewhat tragic how instructors using inclusive instructional practices is an outlier for the Queer students in this study.

Participants described different non-academic spaces with various levels of comfort and feelings of inclusion. When there was a feeling of anonymity within the environment, Queer students viewed these as more inclusive. For instance, large academic study spaces and libraries were described as being more inclusive because everyone is working on something individually and Queer identity is not salient. In contrast, the gym evoked negative experiences and strong feelings of exclusion from every participant because of the people who inhabit the gym, the decisions about what to wear to the gym, and negative past experiences. Cass (Lesbian Cis-woman) and Chase (Pansexual Non-binary) reflected on how there are norms around what “guys” and “girls” wear to the gym and how this can be challenging to navigate. Cass shared “I’m not wearing what other girls wear so that kind of makes me feel weird [because]... my Queerness is very obvious when I go to the gym.” Similarly for Chase, “All of the guys sort of look the same, wear the same thing. The girls sort of you know, always in leggings and so it just feels really weird because I never really know…” The exclusionary nature of the gym we posit is also because of the embodied nature of this environment that appears hostile toward the performance of Queerness.

### 4.2 Psychological identity calculations

Queer students performed various psychological identity calculations after evaluating the environment to then inform behavioral actions. Queer students described psychological identity calculations related to the perceived relevance of Queer identity to STEM, the personal importance of their Queer identity, possible benefits of Queerness within STEM, and fears or risks of Queerness in STEM.

#### 4.2.1 Determining the relevance of queer identity in STEM

Most participants in this study expressed beliefs that their own Queer identity was irrelevant to STEM, but this varied by context. This theme differs from the contextual discourses about STEM, as it shifts from the environment to the internalized beliefs about Queer identity – which is often a cyclic nature enforced through those exclusionary discourses. For example, Emma (Lesbian Cis-woman) discussed that in her computer science lab there is an intense focus on coding and not much dialog at all so her Queer identity does not feel relevant *to her* in those particular spaces. In Horseshoe Crab’s (Lesbian Non-binary) lab experiences she also felt that her Queer identity was irrelevant while working in groups, and she chose to work as fast as possible to get finished and out of the space. Some participants described a desire for their Queer identity to be more relevant in STEM, but ultimately personally minimized its relevance in STEM contexts. Cass (Lesbian Cis-woman) stated, “I’m just working...unfortunately my Queerness gets pushed to the side.” Niko (Aromantic Non-binary Trans-man) and Chris (Queer, Agender) also emphasized a personal focus on their work and learning that minimized how they felt about the relevance of their Queer identity in STEM spaces. Chris shared, “Being Queer is a large part of my identity, so I do not want to hide it, but as a student just learning course material it’s not important.” These participants’ statements further exemplify how Queer students often believe or internalize ideas that their Queer identity is irrelevant in STEM.

#### 4.2.2 Positioning the importance of queer identity

How individuals positioned the importance of their Queer identities impacted how they navigated spaces in higher education. Most individuals expressed the importance of their Queer identities citing that their Queer identities were “a very large portion of who I am,” “important to me,” and “a major part of who I am, who I’ve become.” The participants who explicitly recognized the importance of their Queer identity were motivated by internal validation and less affected by a lack of external validation from non-affirming environments. Sheldon (Gay Cis-man) defined internal validation as “being grounded in that identity” and his success in navigating higher education was accomplished by grounding himself in his Queer identity, recognizing the importance it holds for himself. He attributed his success in Electrical Engineering to his “own self-confidence, instead of trying to get external validation.”

Similarly to Sheldon, three other participants expressed how the personal importance of their Queer identity was a form of internal validation. Niko (Aromantic Non-binary Trans-man) described their Queer identity as “important to me” and continued to say, “I never felt bad about being Queer.” Phin’s (Gay Cis-man) discussion of self-acceptance positioned his Queer identity as an asset he wanted to share with others, “I was like this is me this is who I need to show to everyone.” Blaise (Gay Non-binary) similarly shared that their identity feels relevant and important to themselves and recognized that impacts their interactions with others “...in general, I feel like it is who I am as a person, so I feel like everything should be relevant to people…like they identify this way so that way they do not try to be offensive at all.” Although Phin and Blaise both discussed the importance of sharing their Queer identities with others, they were motivated internally by their own self-acceptance and strength in identity formation. By positioning their Queer identity as something that is important to themselves, participants are removing the opportunity for non-affirming spaces to externally dictate the importance of their Queer identities.

Four of the participants recognized that to be fully accepting of their own Queer identities and see it as important they had to overcome internalized homophobia; homophobia created by external forces relaying hateful messages about Queer identities and not questioning heteronormative views. Horseshoe Crab (Lesbian Non-binary) described her coming out by saying, “I had the internalized homophobia… of you still have to be straight, but also, deep down, you still like girls.” Horseshoe Crab’s internalized homophobia caused her to question her gender as well, citing “it would definitely be a lot easier to meet, date, marry other women if I was a boy, but again that’s not me.” Sheldon (Gay Cis-man) had similar experiences recounting that he used to “hate [his sexuality] so much but then [he] was like well…this is just how it is.” Phin’s (Gay Cis-man) internalized homophobia was created because “everybody was always like [being Queer] is negative,” but eventually they “just did not care anymore” and lessened the influence of the internalized homophobia. The tension of internalized homophobia caused participants to grapple with their acceptance of their Queer identities and impacted how participants viewed themselves. Blaise (Gay Non-binary) specifically described their “journey from not accepting [their Queer identities] to accepting [them as] very mentally tolling.” The internalized homophobia participants experienced imposed an increased cognitive load on participants.

#### 4.2.3 Acknowledging the benefits of queer identity in STEM

Only four of the participants explicitly talked about the benefits of Queer Identity in STEM even with a research design and interview protocol that was intentionally asset-based. The remaining seven participants could not or did not explicitly mention any benefits of Queer identity throughout the entirety of their interview. Chris (Queer Agender), Horseshoe Crab (Lesbian Non-binary), Niko (Aromantic Non-binary Trans-man), and Sam (Questioning Cis-woman Non-binary) talked about how a benefit of Queer identity is in having a “unique perspective” (Chris) where they “have different ways to look at things [and] different ways to solve problems” (Sam) and are maybe “more willing to like look at different solutions for different problems in a sense” (Horseshoe Crab). Horseshoe Crab also took this idea further and talked about how she leveraged her Queer Identity and experience to bolster her arguments in a forensic competition in high school as she:

*could explicitly state that, like as the Queer woman, as a lesbian woman, as someone who’s struggled with gender identity, that these topics are valid and they should be looked at more and more instead of just being pushed to the side for something else*.

Each of these participants shared how they can leverage their Queer Identity to bring a “unique perspective” to the table to allow for “different ways of thinking.” Niko expanded on the benefits throughout the interview stating that the benefit “[is] not just a diverse perspective … [but] it can be easier to [see] where an organization falls short in including other kinds of diversity, when you are one of the kinds of diversity.” This drives home Niko’s belief that “there is a strength to being a Queer person in general that can transfer to whatever field you are in” and that strength as Niko said, is not just specific to STEM.

Several of the participants answered the question about benefits of being Queer in STEM with outright denial. This can be seen in Sheldon’s (Gay Cis-man) response of “Nothing really” or Emma’s (Lesbian Cis-woman) response of “In Computer Science as a female? No.” Phin (Gay Cis-man) on the other hand shared that he “[does not] know if there’s specifically any strengths. I think there’s less disadvantage. But I do not know if there’s any strengths.” These responses by Emma, Sheldon, and Phin highlight that even when asked specifically for the benefits or strengths to being Queer in STEM they cannot identify a strength or benefit. And while Phin explained a strength as “less disadvantage” what he is identifying is still a disadvantage of “feel[ing] like an outsider.”

#### 4.2.4 Fearing the impact of queer identity in STEM

Each participant who was asked, “what sort of fears do you have while being a Queer person in STEM?” responded with at least one fear. The majority of participants referenced “the general fear of just doors being closed to me” (Niko), “[that] they will not accept me” (Horseshoe Crab), “discrimination” (Sheldon), “[being] judged or treated differently than the other people” (Phin), “people being more rude” (Chris), “prejudice, being judged” (Cass), not wanting “people to either ignore me or treat me any differently” (Sam), and “doing any sort of research or anything, then people might try to discredit me for it because of who I am as a person” (Blaise). To capture the unique insights of the participants, it is important to recognize the variations of their fears. As such, we identified four salient fears about the impact of a Queer identity in STEM, which included: fears of changing relationships, concerns of safety, being viewed as incompetent, and fears of being stigmatized.

Four participants discussed their fear of changing relationships when interacting with peers who learn about their Queer identity. Sam (Questioning Cis-woman Non-binary) expressed her fear of working with individuals in her Physics lab, describing it as “a little bit daunting” to interact with “all these other people that you are going to be with for likely the next couple years who I do not know.” Horseshoe Crab (Lesbian Non-binary) discussed past experiences of disclosing her Non-binary lesbian identity before entering university and those peers “not enjoying doing work with me or..stopped communicating” but through current experiences at their university that has not occurred so they are feeling more included. Sheldon (Gay Cis-man) described experiences with homophobic classmates that led them to not trusting the person moving forward. Determining the safety of a peer and worrying about changing relationships is an impactful psychological practice for Queer individuals that is linked with mental health and trauma (Winfrey and Perry, 2021). For instance, Niko (Aromantic Non-binary Trans-man) feared that their “Queer identity being taken negatively probably impacted [their] mental health.” The psychological processes of sorting through unknown relationships allows the participants to sift through people to determine, as Sam (Questioning Cis-woman Non-binary) described who “will not murder [them] on site” and avoid making “an enemy for the next four years.” The fear of going from a neutral peer relationship to enemies or a friendly peer relationship to neutral impacted how participants psychologically prepared to interact with their peers.

Participants often intertwined the fear of changing relationships with concerns of safety when discussing their Queer identities. Most participants alluded to a fear of safety when describing the tension of revealing their Queer identities. Horseshoe Crab (Lesbian Non-binary) elaborated on a time where she “did not feel safe enough to [reveal her Queer identity]... since this was the first time meeting” meeting the person. Sam (Questioning Cis-woman Non-binary) explicitly warns the interviewer:

*You always have to be aware of the people around you because half the time you don’t know if you say something to someone or just make an offhand comment, they just go ‘oh you’re LGBTQ’ and then they could flip a switch. If you’re not careful*.

Other fears participants referenced included: “getting attacked in some way” (Niko, Aromantic Non-binary Trans-man), “get murdered by anyone else on our floor” (Sam, Questioning Cis-woman Non-binary), and “I cannot really trust this person anymore” (Sheldon, Gay Cis-man). The threatening language associated with other’s responses to participants revealing their Queer identities provide context for why participants grapple with the tension of revealing their Queer identities and their safety.

Two participants frequently feared being perceived as incompetent or unprofessional in their current majors or in future occupations. Cass (Lesbian Cis-woman) dissected her experience as a Mechanical Engineering major citing that she may “not [be] viewed as competent” or that “they underestimate [her] for being a woman and a lesbian.” Cass’s recognition of her intersecting identities provided insight into her psychological processes surrounding her identities:

*It kind of makes me feel good when the guys ask me questions because it makes me think that they do think I’m capable of this. But, for some reason, even though I know that I am capable, I’m just worried that other people won’t see me as that for some reason*.

Alex (Bisexual Cis-woman) similarly had concerns of being “perceived as less professional” because of her Queer identity and the ways in which “gender expression can be seen as not cookie cutter.” Alex’s fear of expressing her gender identity via outward appearance and how that could be negatively perceived as unprofessional affects how she perceives herself within her future occupation. Emphasizing that “for [the Queer] community [dressing as themselves is] a little more meaningful than [dressing what others would perceive as unprofessional].”

Lastly, seven participants delved into their internalized fears of being stigmatized in the STEM community for their Queer identities. Sam (Questioning Cis-woman Non-binary) compared the stigma of being Queer in STEM as “similar to being a female in a traditionally STEM field” acknowledging that “people are still having trouble getting over that kind of stigma which is ridiculous.” Cass (Lesbian Cis-woman) similarly felt the weight of the stigma of being Queer through the “constant fear that what if [she] meet[s] the wrong person…and [she] reveal[s] that [she is] Queer to them, and they are weird about it.” Horseshoe Crab (Lesbian Non-binary) feared “the stigmas of the normal, they’ll treat me different.” Niko (Aromantic Non-binary Trans-man), Blaise (Gay Non-binary), and Phin (Gay Cis-man) all utilized future-oriented language when discussing assumed stigmatization. Niko said, “I’ve been very fortunate that I have not *yet* encountered [the stigma of being Queer in STEM].” Phin similarly explained, “I feel like currently I do not think I’ve experienced anything negative *yet*” and continued to elaborate on the future, “I guess because of the future I’m sure there’s going to be different things that I have to experience.”

### 4.3 Behavioral actions

Behavioral actions describe the overt or implicit actions that Queer students would engage with as a navigational strategy within their higher education spaces. These actions were informed by evaluating the environment for a sense of inclusion and performing psychological identity calculations to determine the relevance and risk/benefit of disclosure. Behavioral action sub-themes included: *Building a chosen community, Disclosing or shelving Queer identity,* and *Advocating for representation.*

#### 4.3.1 Building a chosen community

One of the behavioral actions that participants utilized was to foster Queer-inclusive environment by building a chosen community (thus allowing them to determine Queer presence when evaluating the environment). Building a chosen community is a concept analogous to chosen family within Queer communities (Jackson Levin et al., 2020). Queer spectrum students described building a chosen community in different ways which included developing friend groups, choosing certain peers to work with, excluding yourself, or choosing to work individually.

Most of the students discussed the importance of developing friend groups that were supportive and then surrounding yourself with them in higher education settings. The terminology of “supportive,” “safe space for me,” “include me,” “comfortable,” and “positive” were often used to describe these friendships. Blaise (Gay Non-binary) described how they, “find a group of people who will try to include me for who I am and they will not pass me off because I’m just a gay person, so I know that I will always have some inclusion.” Emma (Lesbian Cis-woman) described jokingly how they would attend “gay parties” and meet a lot of people who they would then see on campus which would then be an indicator of Queer-inclusion within academic settings. Cass (Lesbian Cis-woman) described this as finding “a really great group of friends, which I’m very, very lucky to have found. You know it’s mostly with my Queer friends. I always feel safe and always always included with them.”

One way of building a chosen community was to actively select the peers you work with in a STEM space. Chris (Queer Agender) captured the active nature of this navigational strategy in the following way in which he would, “seek out the spaces that are already there because finding community and finding strength in numbers can make you more confident and you more comfortable to be your own authentic self, no matter what space you are in, to sum it up, find the spaces, you want, and then make room for yourself if you need.” Horseshoe Crab (Lesbian Non-binary) talked about how in a classroom setting “normally when you break out into those groups you are allowed to choose who you are with so you are probably going to cho’se people you know you can work better with.” However, deciding peers to work with was not universal and Horseshoe Crab described a different orientation when it is a STEM lab setting “I do not care if I’m an individual or if I’m part of the group, I just want to have the work done so I can leave as soon as I can,” highlighting how the space and context can influence what choice the individual chooses to make.

Cass (Lesbian Cis-woman), Sam (Questioning Cis-woman Non-binary), Horseshoe Crab (Lesbian Non-binary), and Sheldon (Gay Cis-man) all talked about how their individual characteristics influenced them into choosing to work individually in academic settings. For Cass, choosing to work as an individual was because she is “kind of a shy person …[and] not very good at talking to people in a classroom setting.” Sam shared that they “do not have a very easy time talking with most people… [and] so group projects will be very difficult.” Other individual characteristics shared were that they are “very introverted” (Sheldon) and “not a very social person” (Horseshoe Crab) therefore leveraging their agency to work alone when they can. Horseshoe Crab added a layer of complexity to her choice as she “does not generally enjoy speaking with many people because of the past few years and traumas, not always related to being Queer.”

While these four participants framed their agency as choosing to work individually, Phin (Gay Cis-man) frames his agency as choosing to exclude himself. Explaining that “I’m sure there’s been many times where I’ve been excluded or I wanted to exclude myself … especially around straight men, I do not know. I do not always feel comfortable so like if I knew that it was just going to be a lot of them I probably would be like ‘hmmm I do not want to go.” Notice how the language the participants used is very different here as Phin explicItly calls out exclusion by others or by himself versus the language about individuality. Chris shared “I’m usually the most Queer person in the room … I’ve gotten pretty confident and comfortable with it [and] i”s still very obvious that I’m an individual in those settings but like I’ve made myself an individual so I kind of like take it, you know.”

#### 4.3.2 Disclosing and shelving queerness

Participants shared the agency of choice in how, when, and with whom they chose to disclose or shelve their Queer identity. To shelve one’s identity was to not talk about it, to avoid difficult conversations around identity, and or to believe that their identity had no place in a space. Disclosing one’s identity was to choose who they would come out to, to leverage physical appearance clues to Queer identity, and or an expressed desire for those to “just know.”

One of the ways participants talked about shelving their Queerness indirectly was through the avoidance of difficult identity conversations. For Phin (Gay Cis-man) this meant waiting until they were off to college to come out to their parents as “it does not affect me and they can have their little feelings and I will be out of the house.” For others it meant avoiding conversations with peers on campus as “they do not get this quite [and] I did not know if they would understand or not so that gave me a pretty good sign, maybe they would not” (Sam, Questioning Cis-woman Non-binary) or “nobody know how to talk about it, so we just do not” (Chris, Queer Agender). Alex (Bisexual Cis-woman) felt that “it’s just easier to not bring it up” whereas Phin and Niko (Aromantic Non-binary Trans-man) felt that “it’s not really something that should be talked about [in a job]”(Phin) and that the “culture was you did not talk about your partner at work” (Niko). Emma (Lesbian Cis-woman) instead pointed out how the larger community at Hagler University was avoiding the difficult conversations:

*only paid lip service to diversity… they want diversity if it makes them look good but they’re* not going to pursue it because … they don’t want to stir the pot … they’re not going to actually pursue [having more LGBTQ students in engineering] even if they say they want to.

Deciding to disclose their Queer identity to the people around them came down to comfortability for many of the participants. Whether it was comfortability in the form of “the people who I talked to regularly” (Alex, bisexual Cis-woman), specifically friends “I just told my friends” (Chase, Pansexual Non-binary), or it was “a group of friends who were all like kind of LGBT all together which I was just very comfortable around them” (Sheldon, gay Cis-man). Horseshoe Crab (Lesbian Non-binary), Sam (Questioning Cis-woman Non-binary), and Blaise (Gay Non-binary) also talked about how they chose to disclose to those around them that they were comfortable with, whereas Niko (Aromantic Non-binary Trans-man) shared “I am out to most people I know. I’ve said to my partner before that … if someone cannot tell I’m Queer, I am not doing a good enough job kind of thing.” This begins to shed light to another facet in which participants disclose their identity, through physical appearance.

Leveraging the use of their physical appearance whether that be the clothes they wore, the hair style they chose, or the accessories they picked was another common disclosure choice participants talked about. It should be noted that some participants talked about how their physical appearance also made it very obvious that they were Queer and that it did lead to feelings of discomfort, and others often referenced the reactions of others to their action of dressing the way they wished. Chris (Queer Agender), Phin (Gay Cis-man) and Blaise (Gay Non-binary) all talked about “getting weird looks” or “being stared at” due to Phin’s “unusual outfits” or Chris’s “way I dress” or Blaise’s hair as it makes them “a highlighter in a room of pencils.” Phin shared about others assuming their identity as “I mean I go around our own campus walking wearing heels and I do not really care.” On the other hand, Horseshoe Crab (Lesbian Non-binary) shared “when I did first [visit] my physician right now she assumed I was straight and I did not feel like correcting her … everything I was wearing that day kind of screamed gay to me” this highlights how while Horseshoe Crab felt that they were disclosing their identity through their clothes, the physician still made assumptions about being straight. Finally, it is important to recognize how other people in the larger community that dress in similar ways can impact Queer students. Chase (Pansexual Non-binary) said it perfectly:

*I don’t know if they are in the Queer community but they have pink hair and right now, it feels like I’m the only one with purple or colored hair in [academic building] sometimes so just seeing other people with colored hair … makes me feel less of an outcast*.

#### 4.3.3 Advocating for representation

All participants described the importance and impact of Queer advocacy in STEM. Many shared how increased representation would increase their sense of belonging in STEM places and others described more generally how representation would increase their positive experiences in STEM or the experiences of other Queer students. There was general consensus that representation in STEM spaces was currently low with Niko (Aromantic Non-binary Trans-man) summarizing it best, “If you think about where you are going to find Queer people in academia, your first thought is generally not going to be STEM.” Emma (Lesbian Cis-woman) discussed how it is important to see yourself represented in the spaces and described how meaningful it was the first time she saw a character in a movie that shared a similar identity and background as her own. She speculated that the lack of representation of different identities is a key reason for low retention in STEM fields. “They do not feel supported and they do not see themselves in it.” Sam (Questioning Cis-woman Non-binary) also shared that it is important for people who are not Queer to see Queer scientists so they can recognize the competence of Queer individuals in STEM. Niko (Aromantic Non-binary Trans-man) shared a website that they are familiar with called *500 Queer Scientists* that is “aiming to raise awareness of Queer people in STEM where they see themselves represented in STEM.” Emma, Sam, and Niko described a concept that has become a common framework for increasing inclusivity in a variety of disciplines called windows and mirrors (Style, 1988). It is important that individuals are able to see themselves (mirrors) represented in spaces and content and see people with different backgrounds than their own to better understand and appreciate different perspectives (windows).

Some students advocated for representation within course materials, curriculum, and instructors. Chris (Queer Agender) specified how representation of Queer scientists could be integrated in coursework. “It would be amazing if there was an opportunity to show research that’s being done by Queer, gender Queer scientists.” Chris also described how hands-on lab courses can “bring in case studies and identify if the researcher is Queer or non-conforming.” They said, “it’s natural to talk about the research of any scientist so including Queer scientists should not be a big thing.” Knowing that there are other Queer people actively working in the field gives Chris hope that they will find a career in STEM as well. Cass (Lesbian Cis-woman) argued for more Queer faculty. She had an engineering professor who identified as a Queer woman at her previous college and felt that she could relate to her and was more open to asking questions. She experienced a stronger sense of belonging and as a result more meaningful engagement in this course where her identity was represented.

Finally, almost all students reflected on how they themselves could serve as role models and be advocates for the next generation of Queer students in STEM. Alex (Bisexual Cis-woman) shared how she could personally be an advocate by being more vocal about her identity within STEM spaces and in her department. Horseshoe Crab (Lesbian Non-binary) described how she would use forensic competitions in high school as a platform to discuss socio-political issues that impact the Queer community. Chris (Queer Agender) shared that “because my journey to get here was not easy… I feel like I can be a listening ear for other students” and describes being a role model as “being a light for other people.”

## 5 Discussion

Through our analysis in this study, we developed a conceptual model for how Queer STEM students navigate higher educational environments (see Figure 3). We view this model as a cyclical, changing, and contextual framework whereby students are attending to environmental factors, processing psychological considerations, and then deciding on a behavioral action. This process repeats itself as students engage in behavioral actions of advocacy or disclosing their identity, which then impacts their perceptions of the environment, and future psychological considerations. In many ways this model draws parallels between teacher noticing frameworks (König et al., 2022), whereby instructors first must attend to student thinking, interpret the information based on their pedagogical content knowledge, and then requires a responding action from the instructor which could be sequencing content or making student thinking visible. We draw this parallel to teacher noticing to aid the reader who does not share the lived experience of having a Queer identity, to make connections to the fast-paced, quick, and micro-decision making that occurs in instructional practices. Furthermore, professional development is often used as a tool to aid in teacher noticing of asset-based content knowledge in similar fashion whereby Queer students are receiving training and feedback throughout their daily existence within these spaces.

When Queer students are on constant high alert, they describe how the mental load of their Queer identity weighs upon their conscience. Queer students in our study described how they are more acutely aware of their identity when in public or homogenous (white cishet males) groups and have to be more aware of their actions and performance of Queerness (Butler, 2009). Queer students might reveal their Queer identity to others, which can promote a sense of self-integration and personal empowerment (Corrigan and Matthews, 2003; Baiocco et al., 2016); however, the ability and decision to reveal one’s Queer identity is often multifaceted and situational. For instance, Toynton (2016) put forth the notion of Queer identity in STEM as the “invisible other,” such that being Queer-spectrum is an experience of being the “other” and yet invisible if wished. The invisible nature of Queer identity provides agency to reveal one’s identity, while at the same time requiring ongoing decision-making to determine whether and how to disclose this identity. Research indicates that having to navigate coming out in educational spaces creates more emotional and psychological work for Queer students and often results in daily decisions about revealing their sexuality in the classroom (Lopez and Chims, 1993; Eliason, 2019; Savage and Harley, 2009; Toynton, 2016).

Participants in this study discussed the fears about being Queer in STEM and determining if it is a safe environment to share their identities while also conveying tensions of internalized homophobia and questioning the relevance of having a fully humanized self within STEM. Research has shown that sexist attitudes correlate with internalized homophobia or homonegativity (López-Sáez et al., 2022), thus STEM environments may exacerbate these psychological functions given the fields history of sexist attitudes (Saxena et al., 2019), exclusionary practices (Hennessey, 2018), and history of misogyny (Rutherford, 2020). When individuals do not feel as though their identity is relevant or accepted in a space, they will often shelve their queerness to avoid uncomfortable or unsafe situations. In this study, participants downplayed moments of marginalization by following stories of perceived marginalization with a comment that questions whether marginalization took place or minimizes the severity of the marginalization. By downplaying marginalization, participants are attempting to mentally protect themselves from the negative treatment of others. This psychological practice could be harmful to participants as they are taking on the burden of reframing their own negative experiences.

Participants in our study described a desire for increased Queer representation in STEM environments and the hope that others infer their Queer identities based on their appearance or physical presentation. Appearance and the use of symbolic interactionism (Stone and Farberman, 1982; Hutson, 2010) is a growing area of interest in Queer identity research, with some suggesting that appearance and dress are one of the primary mechanisms for ascertaining and displaying such identities. The use of dress or appearance can serve to create a sense of group belonging in Queer communities, resist normative gender expectations, express authentic self-identity, and signal their identity to other people “in the know” (Hutson, 2010; Rothblum, 2014). Furthermore, appearance by Queer individuals is a process of negotiation that is impacted by the environment and the current socio-political context. For example, indicators of Queer identity status have shifted over time from more coded indicators in the “era of the closet” (Seidman, 2002) using colored handkerchiefs (Reilly and Saethre, 2013), fashion brand logos (Clarke and Turner, 2007), to more explicit indicators and gender-Queer fashion (Barry and Martin, 2016) in the post-closet era.

Queer identity can also be marginalized and oppressed within educational environments even without students disclosing their Queer identities. These marginalizing forces occur through the presence of microaggressions (e.g., derogatory statements, invalidations, insults) that creates barriers for students in coming out (Vaccaro, 2012; Vaccaro and Koob, 2018). For instance, 99% of Queer-spectrum youth report hearing the derogatory use of phrases such as “that’s so Gay” or “you are so Gay” in school (Kibirige and Tryl, 2013). These forms of oppression help align education with heteronormative experiences and have been shown to result in higher rates of depression, substance abuse, social isolation, and suicide (Herek et al., 2009).

Although we used Queer to refer to students that identify as LGBTQQIP2SA+ we understand that there are several important differences in identity and experiences that are not captured in our data and analysis as a result of combining them into one group. As such, we recognize the limitations with this approach such that: individuals with the same identity (e.g., Pansexual) have different lived experiences, Queer can consists of both gender and sexual identities, variability within the Queer spectrum for both more visible and hidden identities, and the themes described in our results may be experienced differently by each participant especially considering intersectionality with other forms of oppression. Chase (Pansexual Non-binary) illustrates this when describing a campus organization for Queer students, “I just felt a little left out like there wasn’t something for people under the Trans umbrella, and because our experiences can be at times a lot different than other people in the LGBT community.” These differences are important and should be studied more extensively, but the commonality is that Queer students in STEM are “managing their identities in learning spaces that are heteronormative, gender normative, and historically heterosexist” (Reynolds and Hanjorgiris, 2000).

Our analysis spotlights how Queer identities are not well represented in STEM learning spaces. This is consistent with findings from Levya et al. (2016) that the technical nature of STEM disciplines is prioritized above social aspects (e.g., technical-social dualism) which often alienates those who do not identify as white, cisgender or heterosexual. This also highlights the need to transform STEM pedagogy to be more “exploratory, fluid, and open” (Voigt, 2020) thus leveraging a Queer theory perspective to re-imagine STEM. We close this manuscript by sharing what participants in this study recommend to support Queer students in higher education at large, STEM disciplines specifically, and in general day to day life. Overall, students advocated for the proper use of pronouns by instructors, highlighting Queer scientists in curricular units, increasing inclusivity by talking about identities in classrooms, supporting identify-affirming organizations (e.g., oSTEM), and increasing representation throughout the field. These recommendations drawn directly from students are not surprising and align closely with prior research studies (Cooper and Brownell, 2016; Hughes, 2018; Kersey and Voigt, 2021). Building on these results, we developed a tiered instructional strategies guide (see Appendix B) that may support instructors in designing learning spaces that are more inclusive and promote more equitable outcomes for Queer-spectrum students.

As we conclude, a fundamental question arises: when will we take action to disrupt the oppressive structures in education and alleviate the trauma experienced by Queer students in STEM? We fear that if we do not take quick and decisive action, the forces that are committing genocide against individuals who are transgender, the murder and brutalization of Queer bodies, and the denigrating rhetoric that silences voices of opposition will rise to levels that have not been seen in recent history. This is a plea to readers and the academic field, that many members of the Queer community are experiencing fear and trauma for our safety, and this cannot be normalized. We close by sharing the inspiring words of two prominent Queer advocates. The first is from Marsha P. Johnson, a leader of the Stonewall uprising, “History is not something you look back at and say it was inevitable. It happens because people make decisions that are sometimes very impulsive and of the moment, but those moments are cumulative realities.” The last quote comes from Audre Lorde, “When I dare to be powerful — to use my strength in the service of my vision — then it becomes less and less important whether I am afraid,” and “we are powerful because we have survived.” So, we implore the reader to make impulsive decisions without fear in order to support a vision of a just and inclusive world for Queer individuals.

## Data availability statement

The datasets presented in this article are not readily available because the data contains sensitive information and maybe re-identifiable given the rich nature of the interview data. The raw data from this study may be made available upon request after providing a written rationale and intended use of the data. Requests to access the datasets should be directed to MV mkvoigt@clemson.edu.

## Ethics statement

The studies involving humans were approved by Clemson University Office of Research Compliance. The studies were conducted in accordance with the local legislation and institutional requirements. The participants provided their written informed consent to participate in this study.

## Author contributions

MV: Conceptualization, Formal analysis, Funding acquisition, Investigation, Methodology, Supervision, Visualization, Writing – original draft, Writing – review & editing. MB: Formal analysis, Investigation, Methodology, Project administration, Supervision, Writing – original draft, Writing – review & editing. DC: Formal analysis, Investigation, Methodology, Supervision, Writing – original draft, Writing – review & editing. SO: Formal analysis, Investigation, Methodology, Supervision, Visualization, Writing – original draft, Writing – review & editing. AS: Conceptualization, Formal analysis, Investigation, Methodology, Writing – original draft, Writing – review & editing. CW: Formal analysis, Investigation, Methodology, Writing – original draft, Writing – review & editing. CH: Conceptualization, Formal analysis, Investigation, Methodology, Writing – original draft, Writing – review & editing.

## Footnotes

^1^Hagler was a chief of the Catawba Native American tribe from 1754 to 1763. We choose this pseudonym to recognize the ways in which the ancestral lands were stolen from native communities and given under the auspices of “land-grant” universities.

^2^A phrase from the Southern United States meaning, to get along.
