# Supplementary material for: Building a model of navigational strategies for queer undergraduate students in STEM
Source: Front Sociol. 2023 Nov 30;8:1293917. doi: 10.3389/fsoc.2023.1293917 (PMC10720439; doi:10.3389/fsoc.2023.1293917)

Supplementary Material

Appendix A

Queer in STEM Individual Interview Protocol

**Introduction**

| Pseudonym: | Identity: | Pronoun: | Major: |
| --- | --- | --- | --- |

Thank you again for your willingness to participate in this study, as you may remember, this is a single interview and will last roughly an hour. I am interested in hearing your story and background as someone who has a queer identity while pursuing a STEM-related major at Clemson.

Anything you share with me today will be anonymized in my write-up, and I will send it to you to check-over to make sure I captured what we talked about accurately.

*[have student review informed consent and affirm recording]*

*[Instruct the to change the name on zoom with pseudonym -- three dots on profile then rename]*

The goal of this interview is to be as conversational as possible, but I do have some guiding questions and topics to structure our conversation

Before we start, do you have any questions for me?

**Identity**

1. The aim of this research is to understand and advocate for the experiences of LGBTQIA+ or queer-spectrum students in STEM fields. Because Queer and STEM are such broad terms, can you share how you personally identify with being LGBTQIA+?
   1. What does _<identity label they choose>_____ mean to you?
2. Are there other important identities or communities that you belong to or associate with?
3. What are you currently majoring in and how did you come to select that major? What drew you to a STEM field as opposed to a different discipline?

**College Experience**

1. What about Clemson appealed to you when deciding on a college? How do you feel about your decision?
2. What sort of resources (e.g., SAGA, STEM clubs, tutoring) do you leverage while being queer in STEM? How did you find out about them?
3. Do you have access to inclusive medical or counseling resources? How does that impact you as a student?
4. What are some unique aspects of being queer that impact your experiences in STEM?
   1. Do you feel like part of a community or more of an individual?
   2. What sort of fears do you have being a Queer person in STEM?
   3. Do you perceive or know of any stigmas about being Queer in STEM?
   4. Are there strengths of being Queer in STEM?
5. Do you see queerness represented in STEM spaces? In course materials? In classrooms? What would it mean to you to see queer people positively represented in STEM?

**Coming Out**

1. When did you first start coming out (to yourself or others)? What was that journey like for you? Are you out to most people you know?
2. How were you received? In School, at home, with friends? Do you feel comfortable being out in your classes or lab space?

**Queer Discourse Exploration Activity**

1. Think about situations in which you have felt particularly included/excluded because of your sexuality, what about those situations made you included/excluded?
2. Can you describe situations in which you are particular aware of your queerness? What about situations where you are least aware of your queerness?

We’re going to describe some types of academic spaces and life events and we’d like you to think about your own experiences and place each of them on a spectrum according to how excluded or included you feel/felt in those spaces. It would be very helpful to us if you could talk aloud about your thought process while you place these.

<Sorting activity>

**Included/Excluded**

**
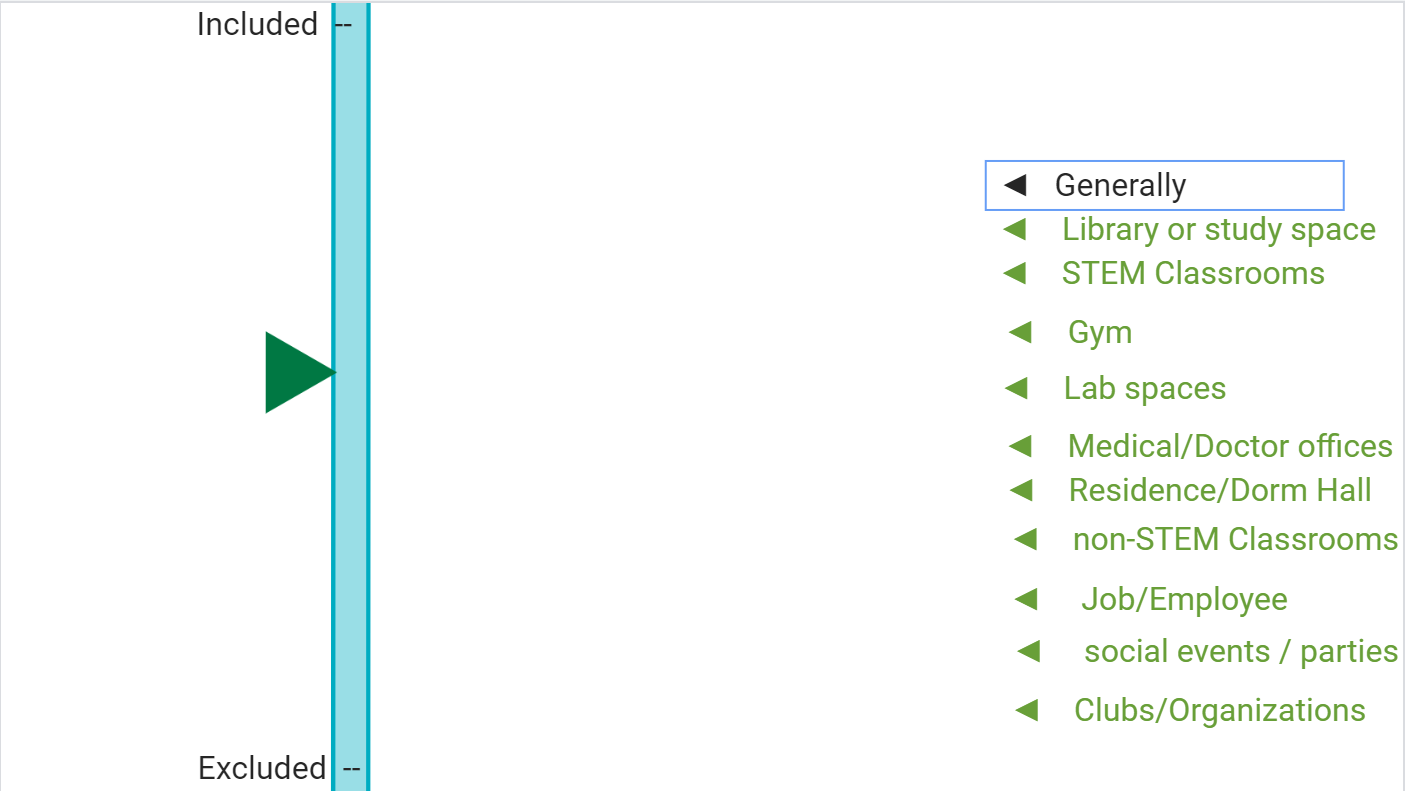
**

**Relevant/Irrelevant**


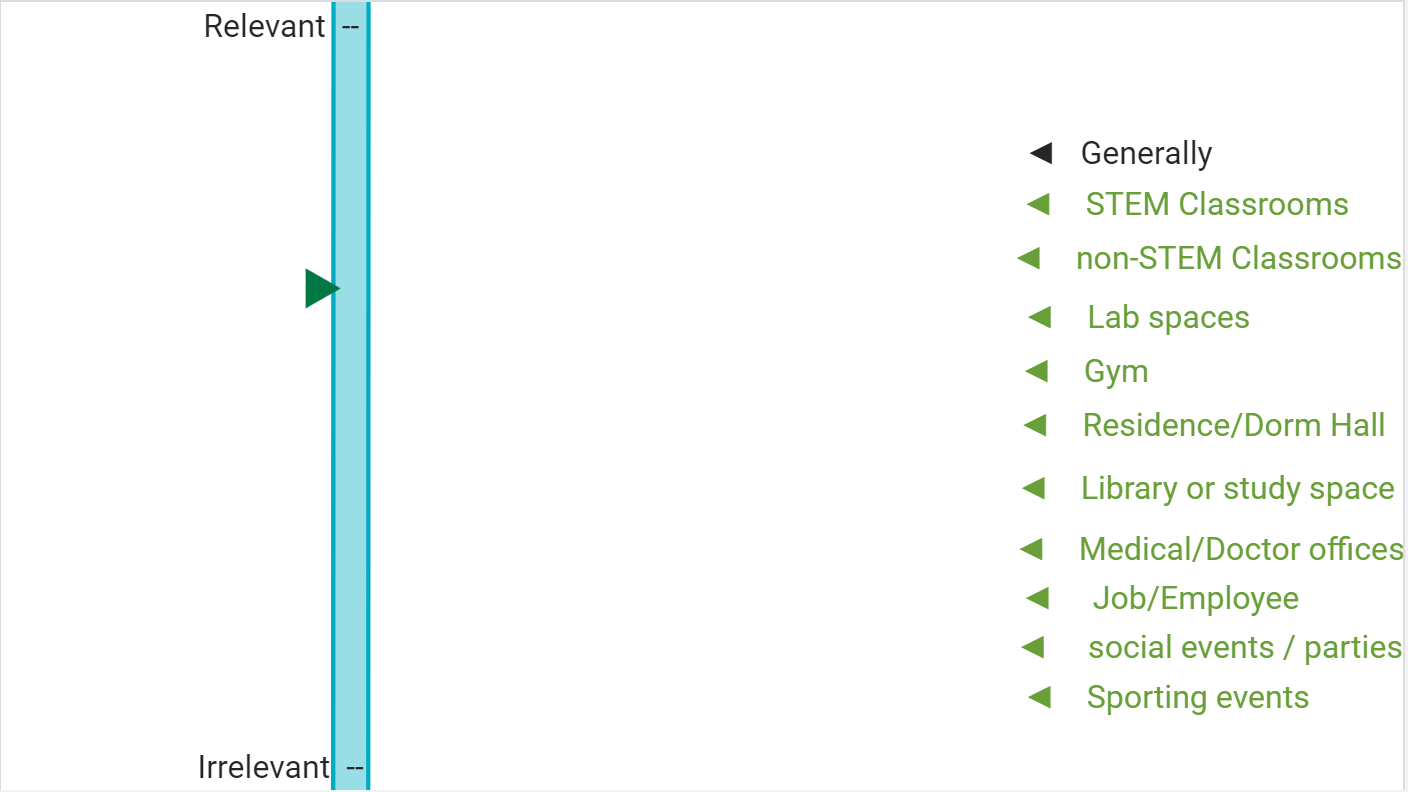


**Inclusion-Relevancy Plane**

Now we are going to pull it all together, to place these spaces and life events on both scales. Once you have them placed we will talk about each of the quadrants.


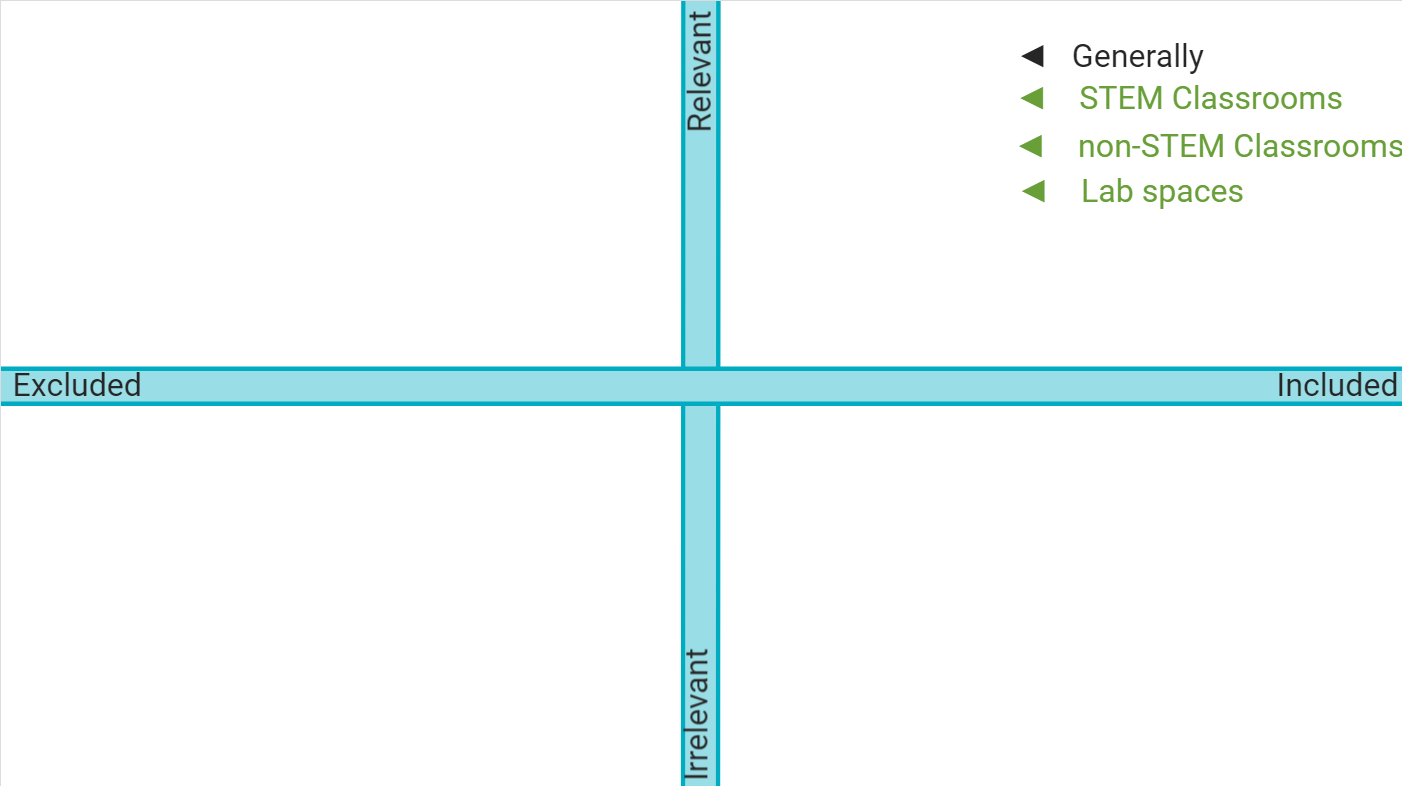


**Closing**

16. What recommendations would you have for others to make STEM spaces more affirming/positive for LGBTQIA individuals?

17. What are three words would you use to describe yourself?

Thank you so much for sharing with me today! I am looking forward to speaking with you again.

Exclusion-Irrelevancy Graphs for Each Participant


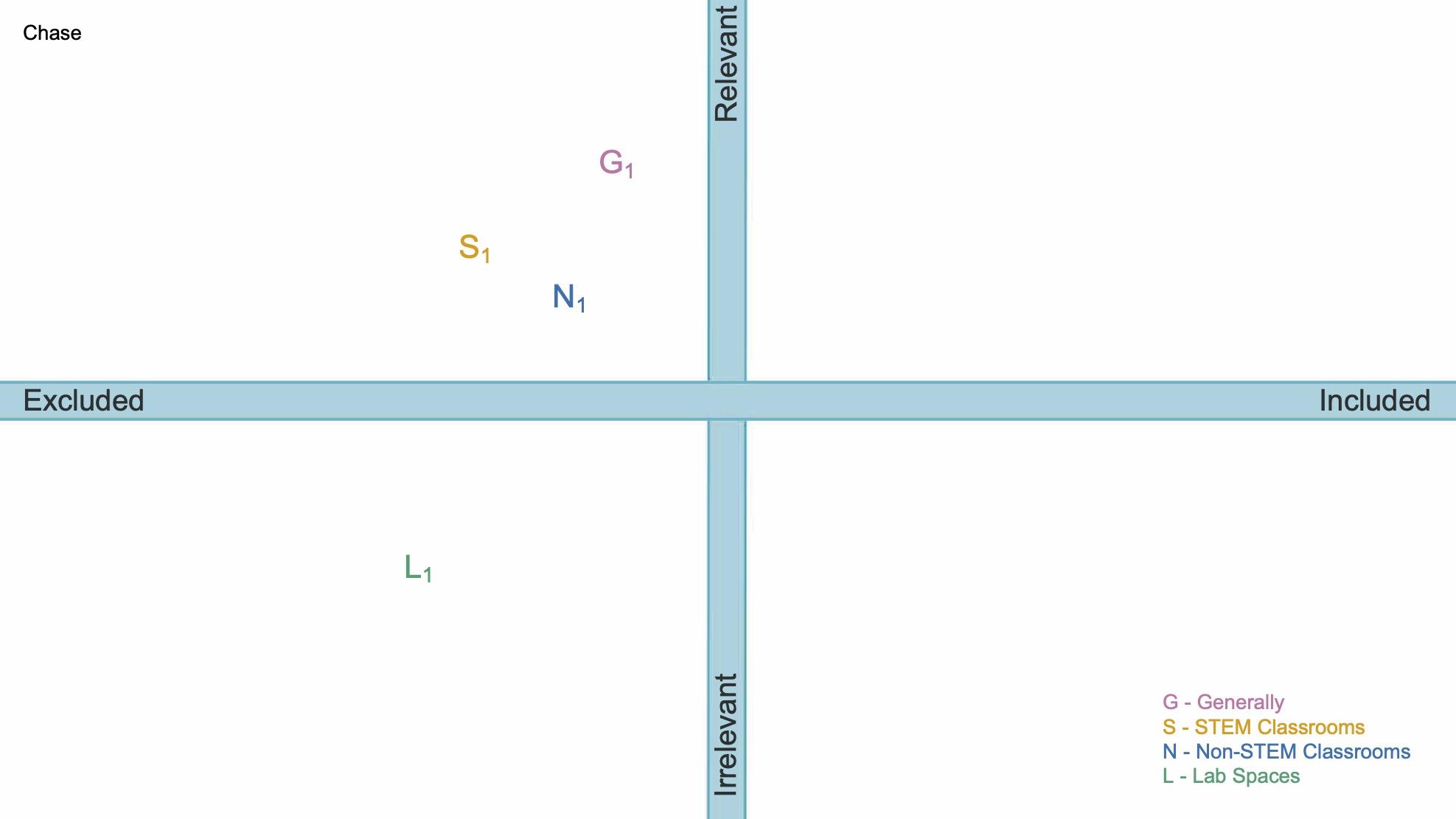


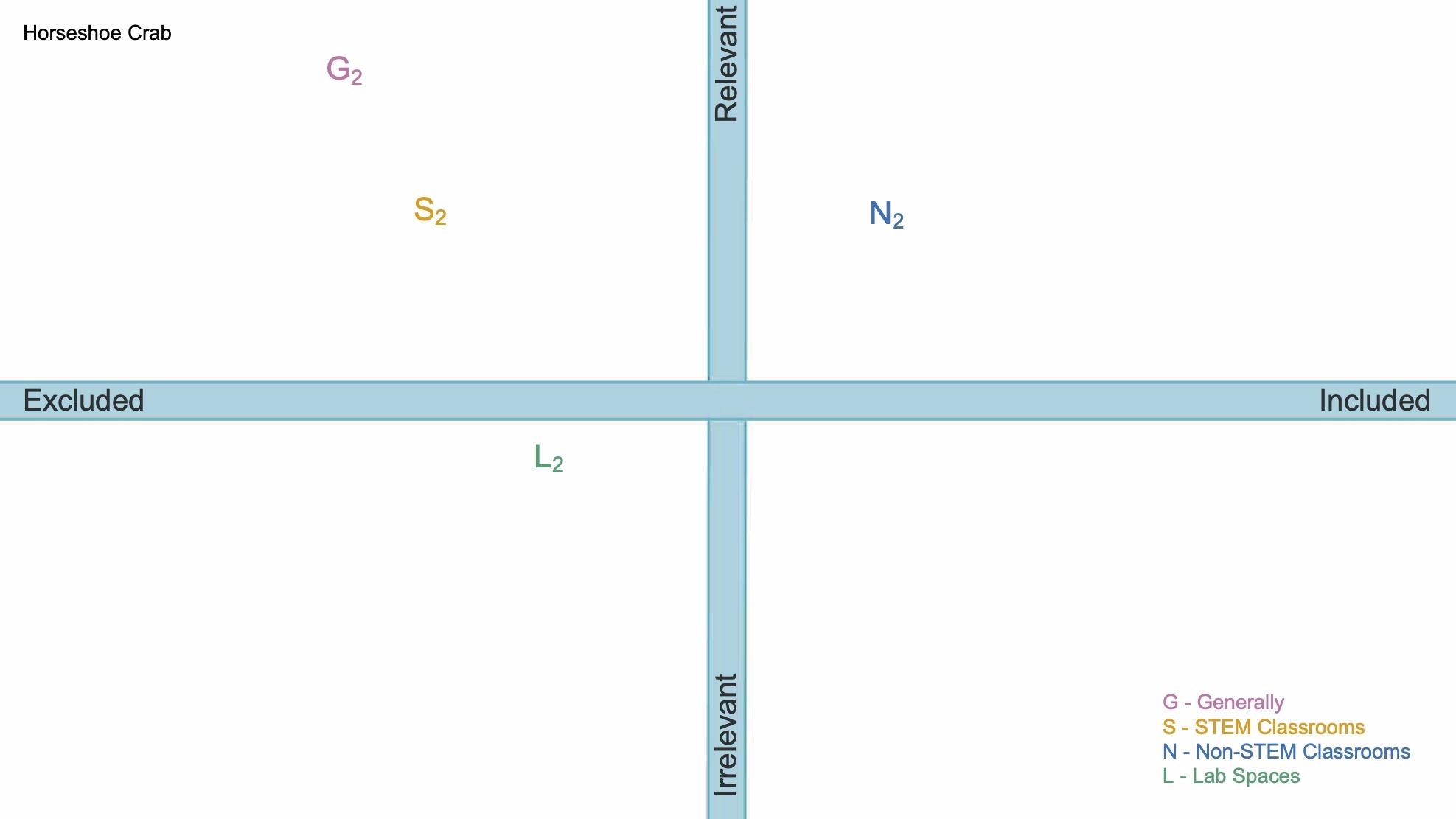


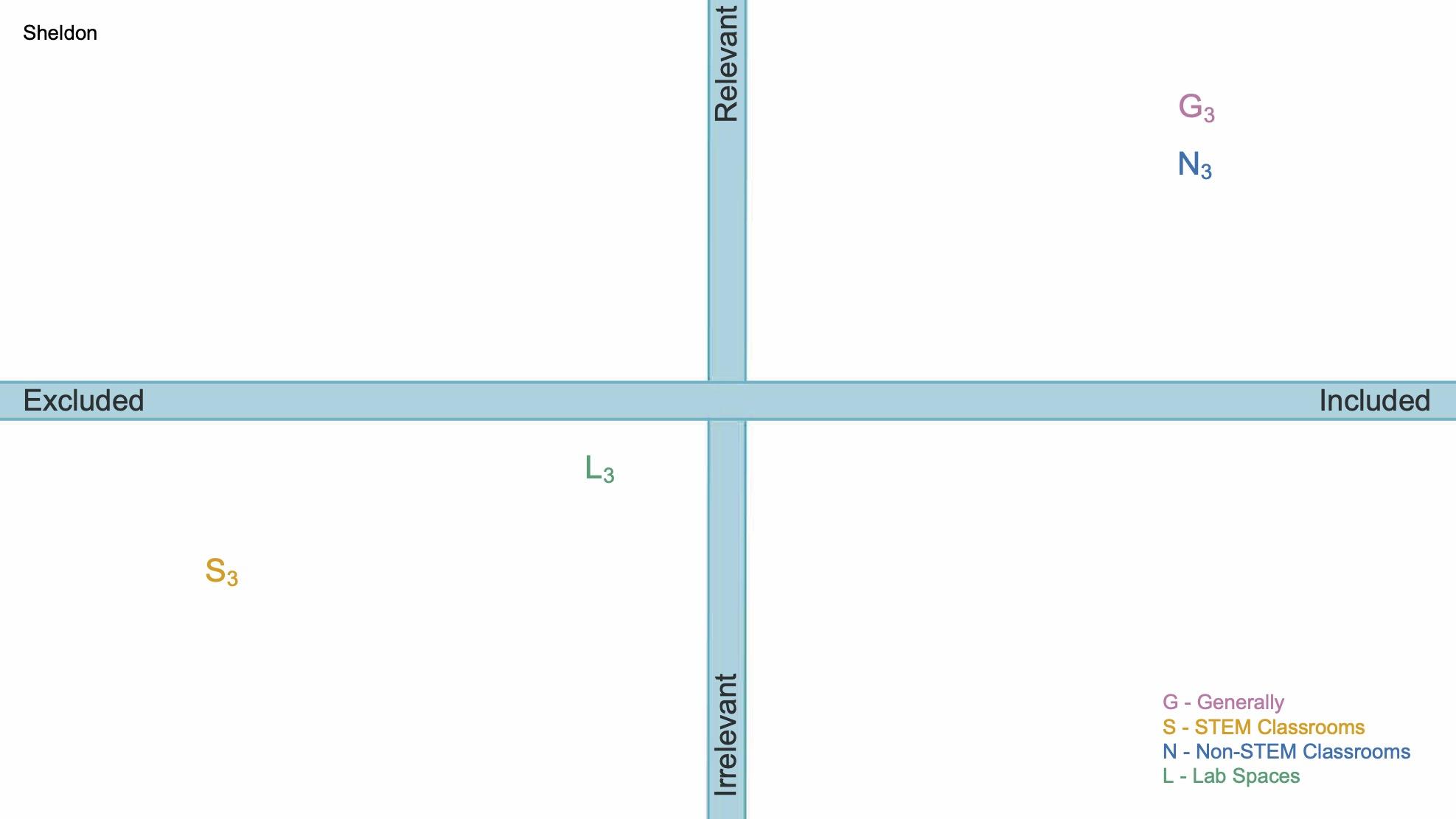


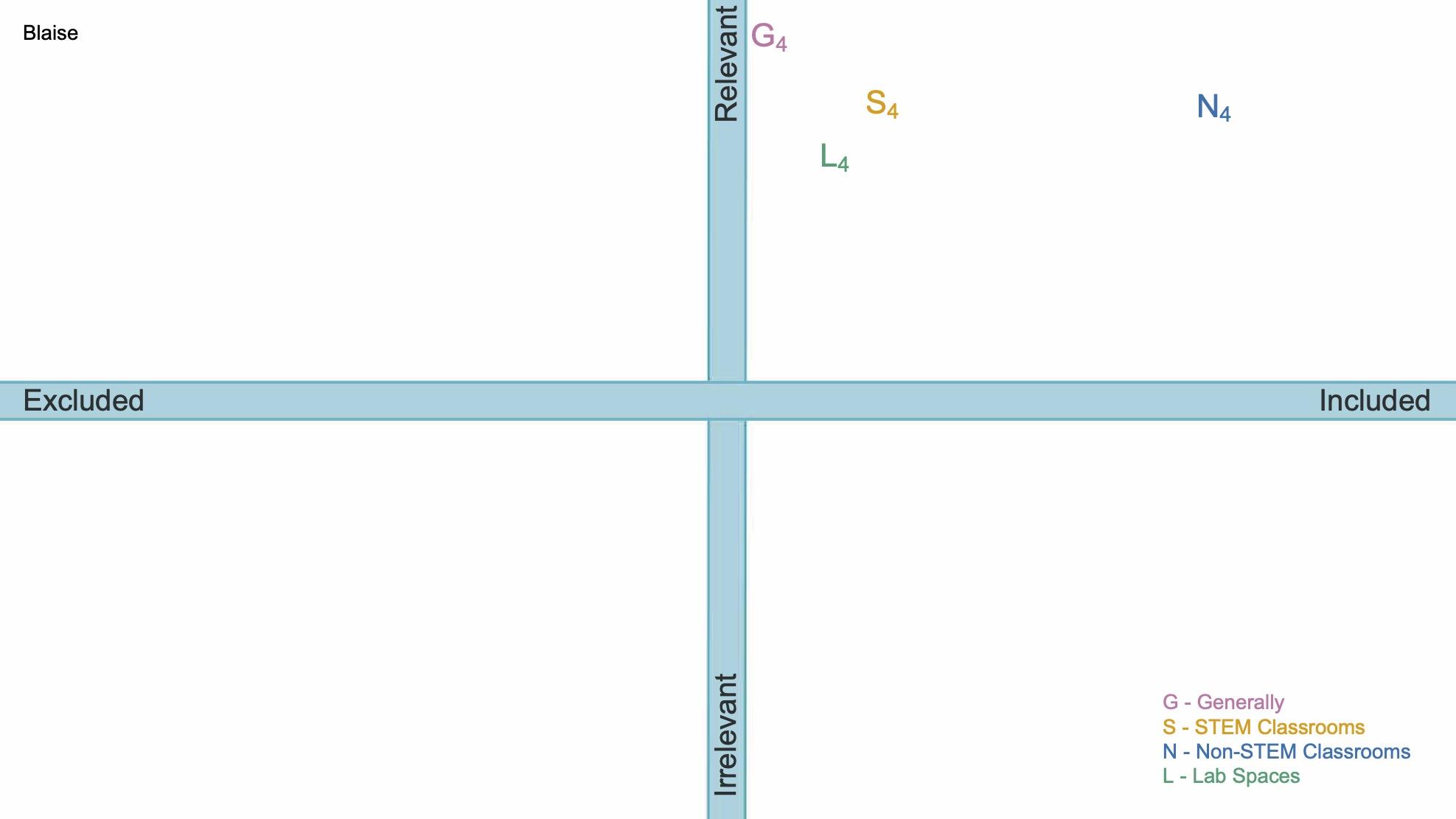


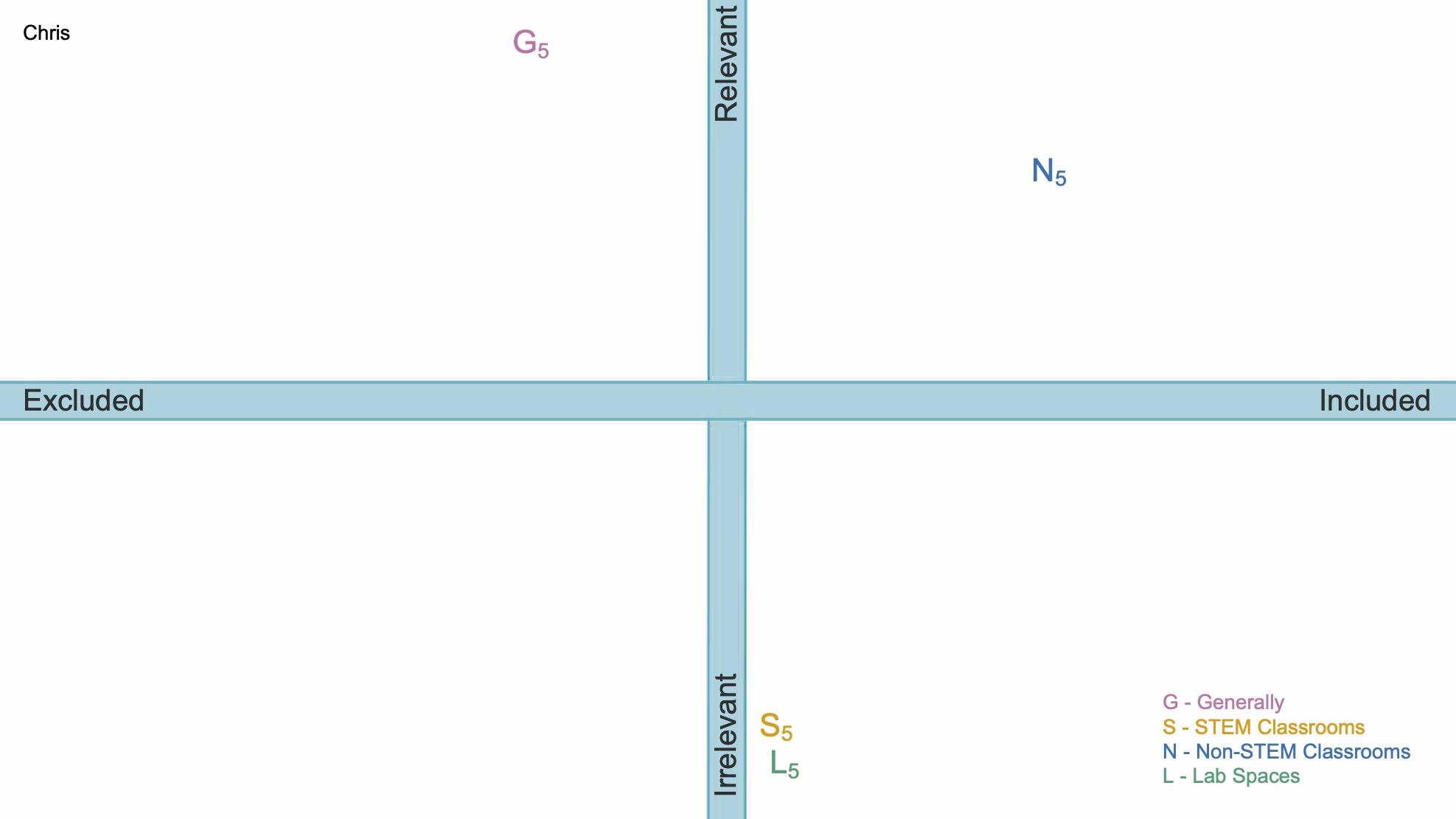


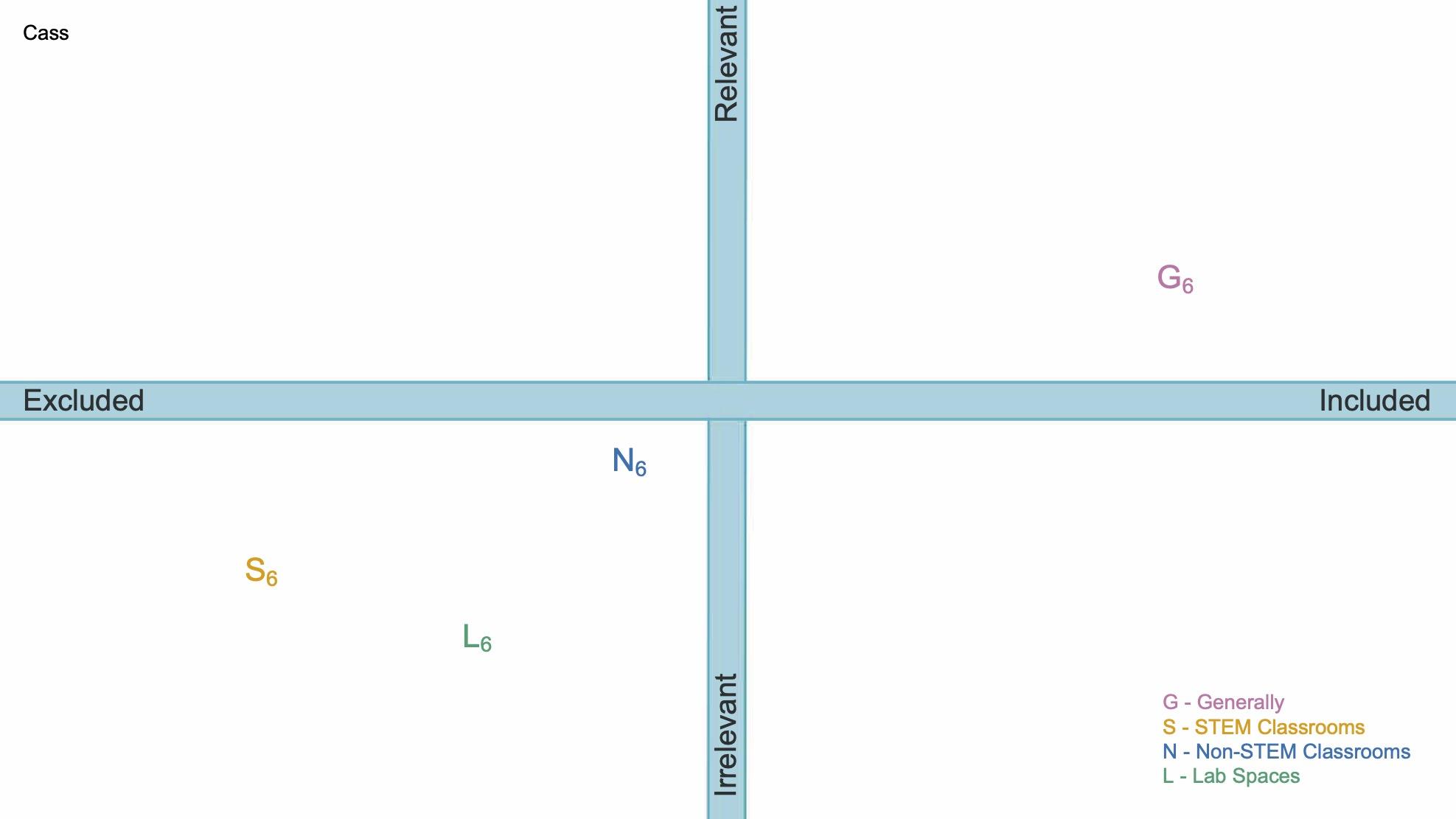


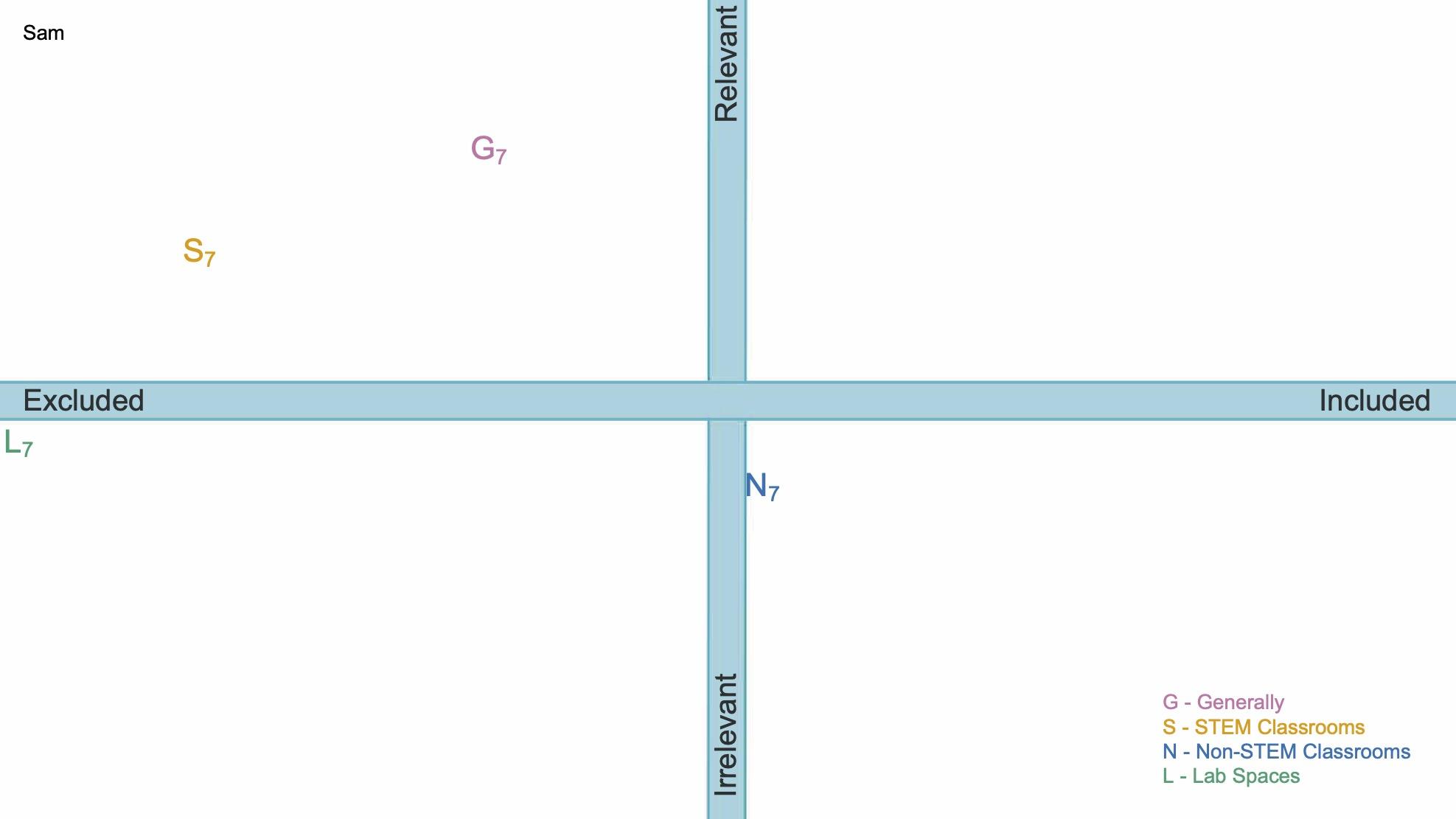


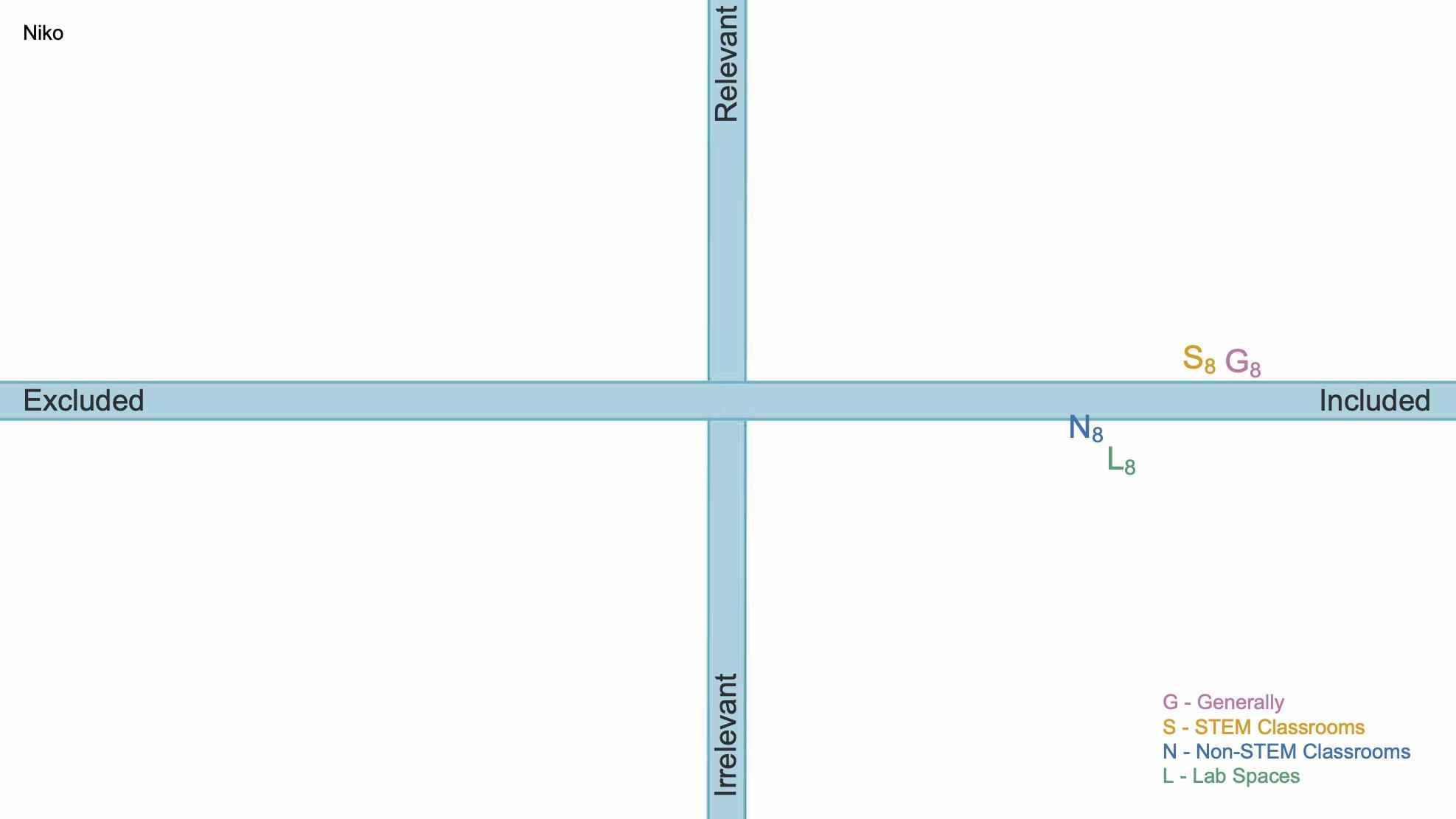


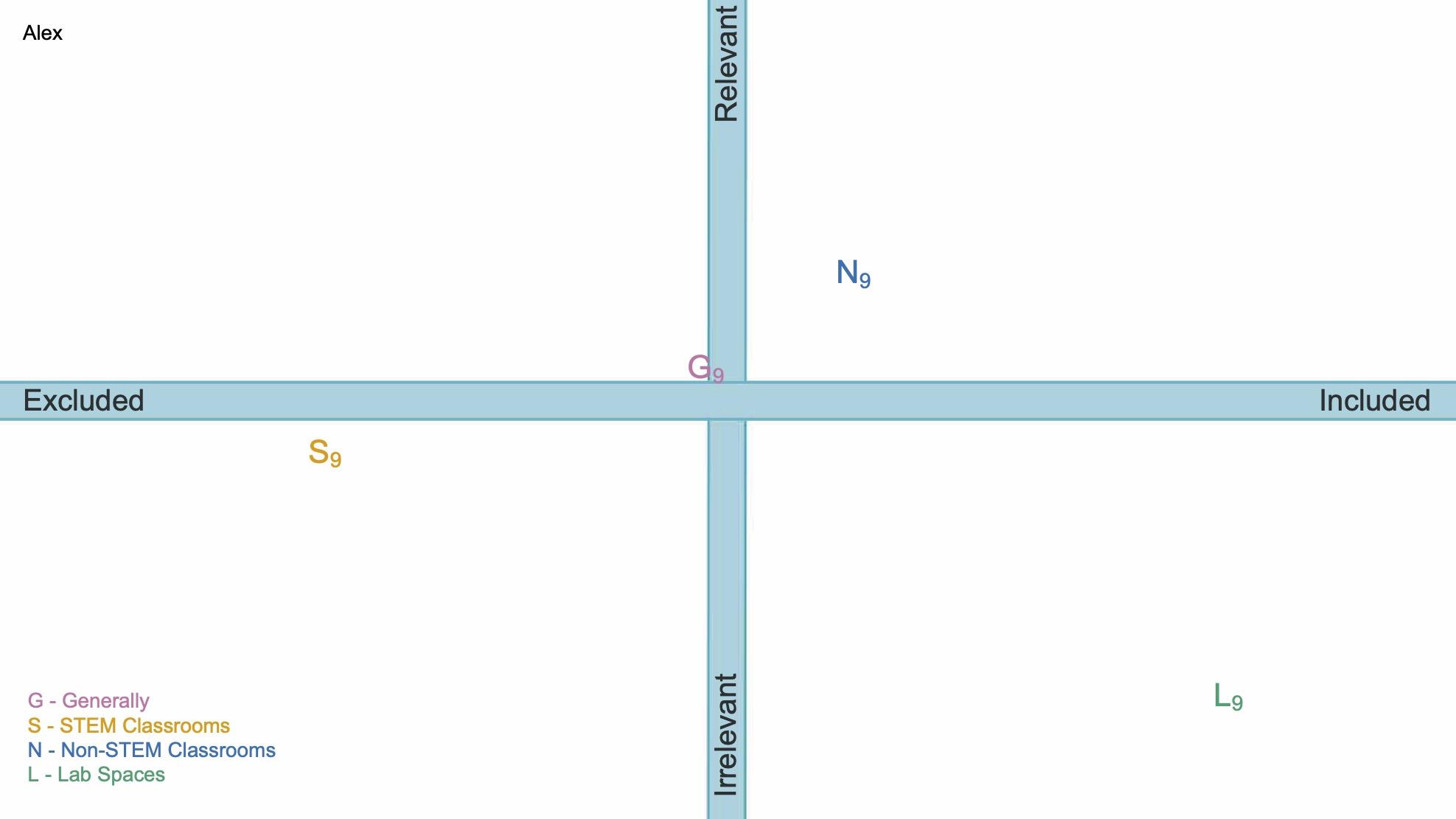


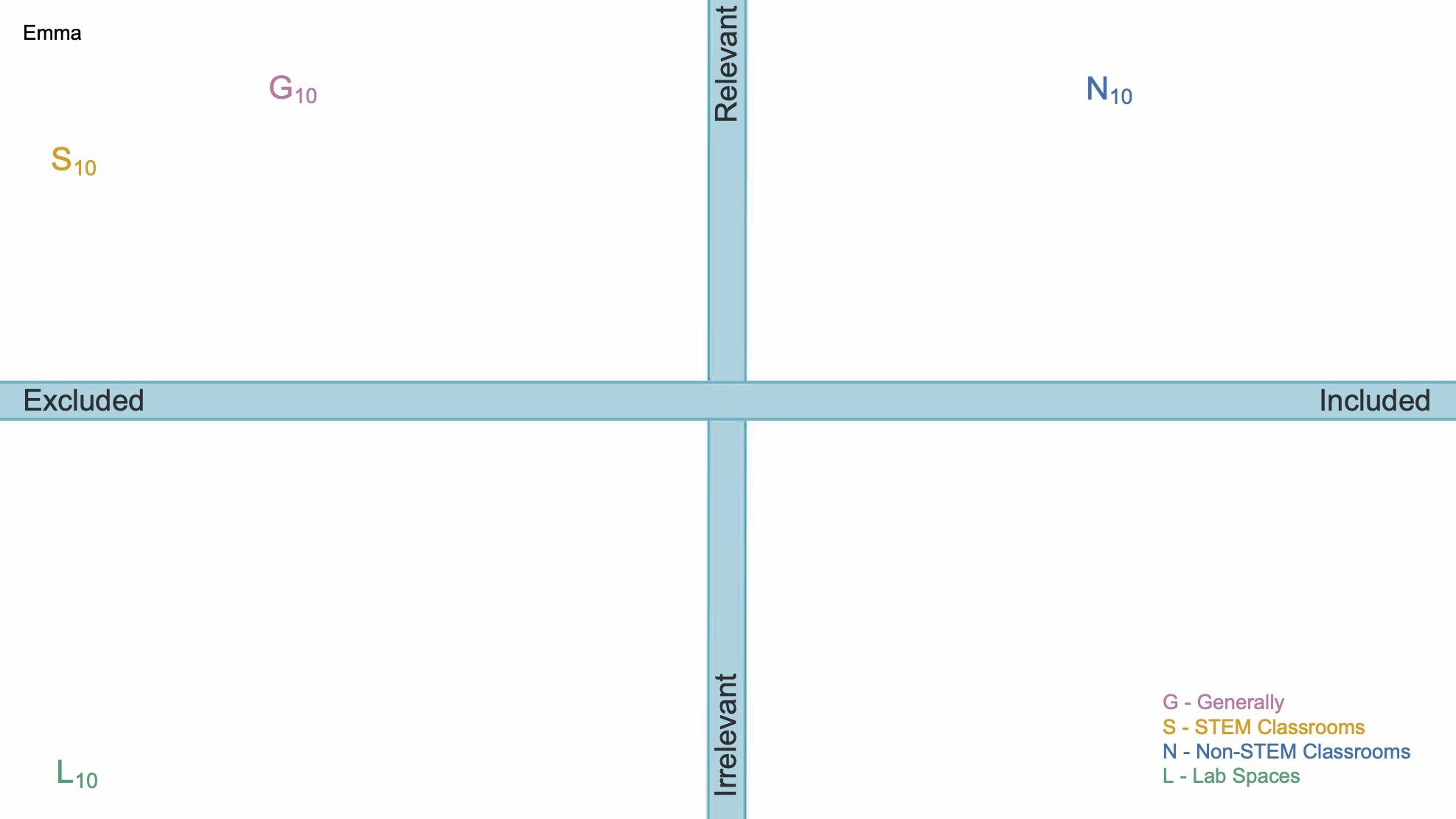


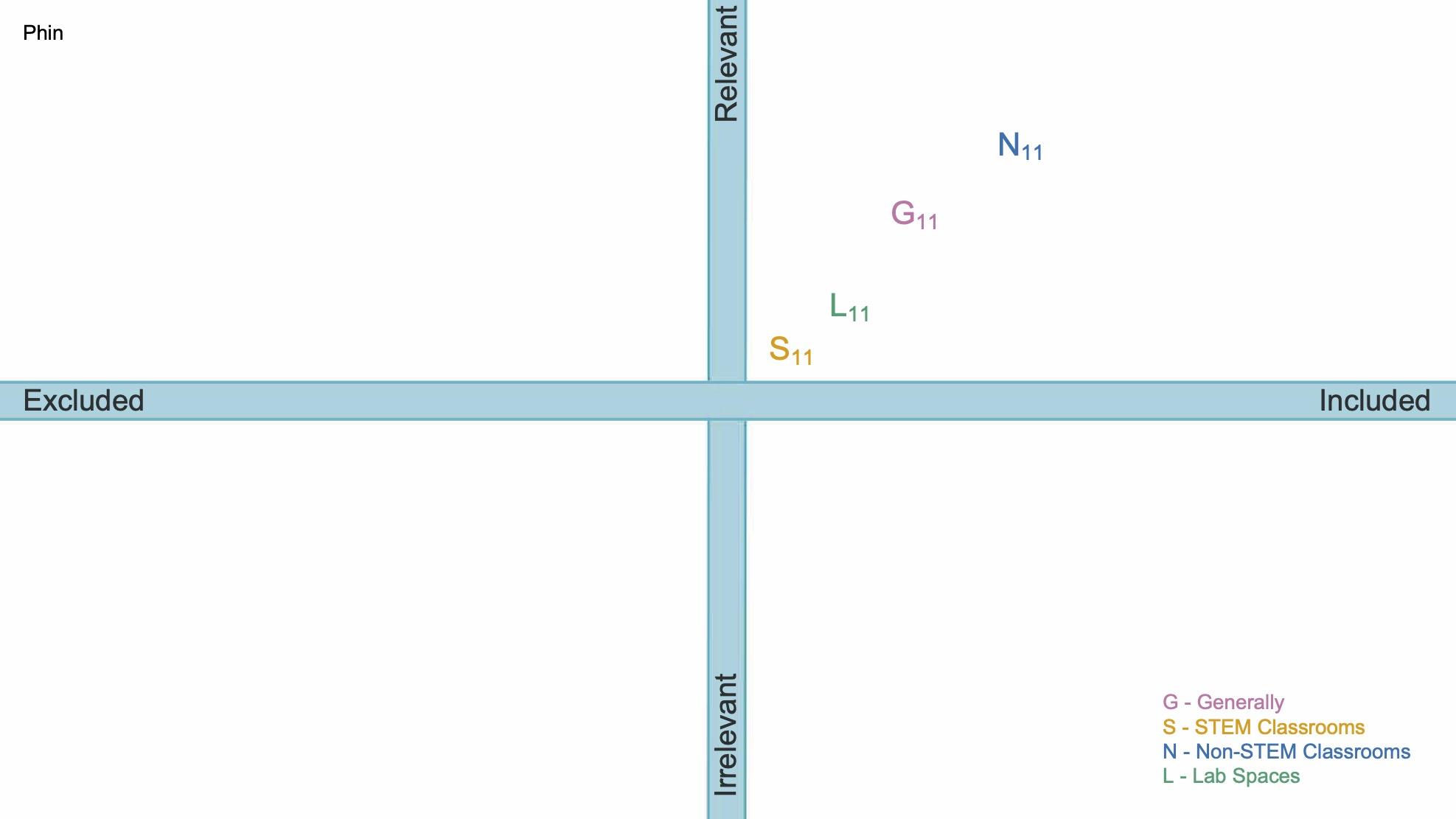


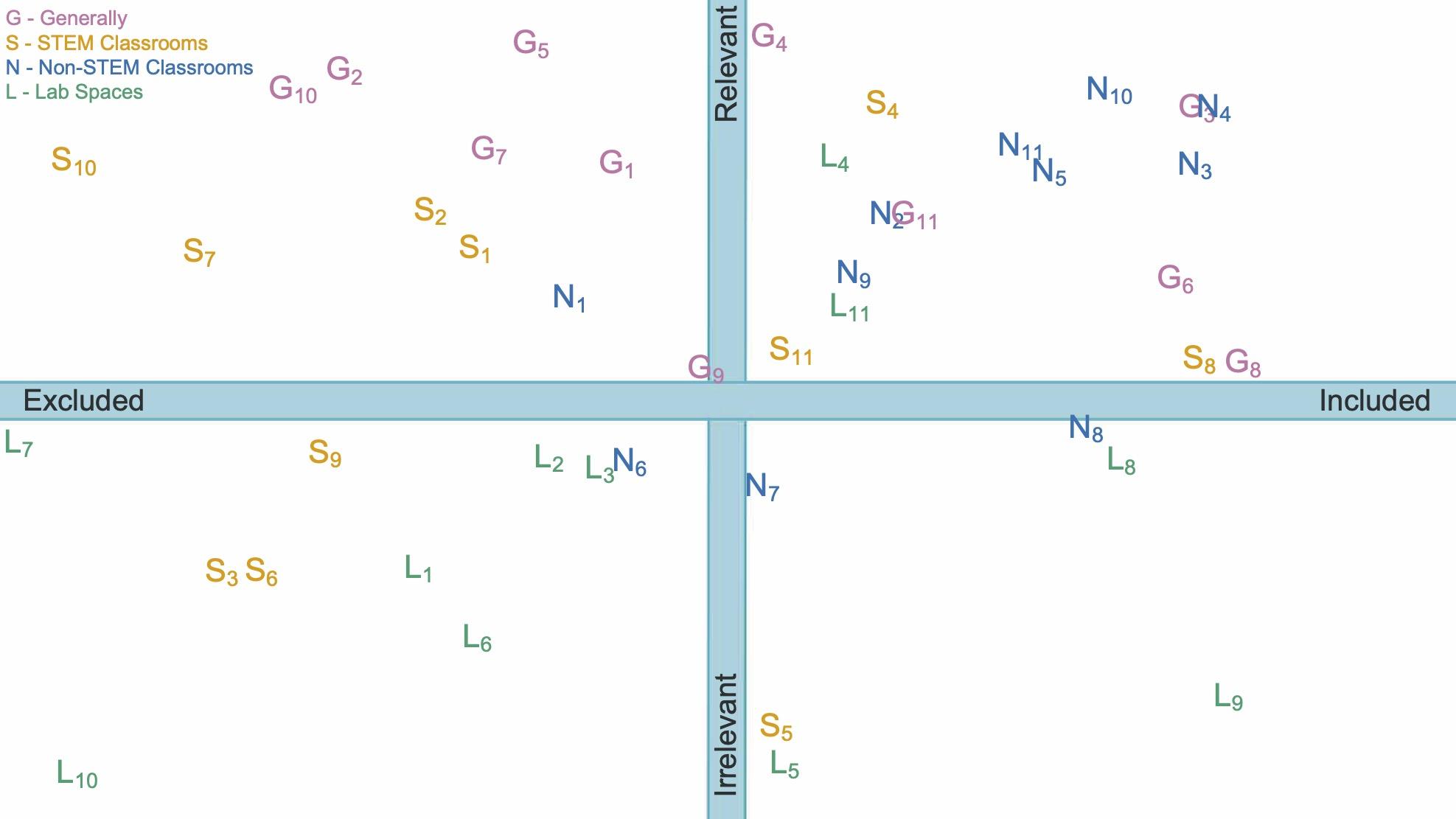

Supplement: Supplementary file 1 [file Data_Sheet_1.docx]
