# Supplementary material for: Building a model of navigational strategies for queer undergraduate students in STEM
Source: Front Sociol. 2023 Nov 30;8:1293917. doi: 10.3389/fsoc.2023.1293917 (PMC10720439; doi:10.3389/fsoc.2023.1293917)
Supplement: Supplementary file 2 [file Data_Sheet_2.docx]

**Appendix B**

**Creating Safe Spaces for Queer Students in STEM**

Dear STEM Colleagues,

In our research focused on the experiences of Queer students, we learned that they describe certain academic spaces as unwelcoming. We understand that there are a lot of factors within the classroom that are out of your control, but there are a few things that you can do now to foster an inclusive environment for all of your students. As a result, students will be more likely to talk in class, ask for help when needed, and work more effectively with their peers.

These strategies are organized into two levels. We encourage you to implement the Level 1 strategies the next time you are with your students, but the Level 2 strategies may require more planning and reflection before you feel ready to try them out.

# Level 1 Strategies

## Signage/Wording/Pronoun Usage

- Incorporate signage to signify a safe space for students.
  - Signs around the classroom
  - Flags (e.g., Country flags, Queer-spectrum flags)
  - Laptop or water bottle stickers
  - Pins for your clothes or bag
- Be intentional about word choice when speaking or creating assignments.
  - Using folks/folx instead of guys
  - Them instead of he/she
- Openly share your pronouns and ask students to share their own pronouns orally or in writing (some students may not be comfortable sharing in front of the whole class).
- Use correct student pronouns throughout the semester.

By providing visuals of support and modeling supportive structures, students will feel more comfortable in the space. These suggestions will increase student feelings of safety, subsequently increase student engagement and improve learning outcomes.

## Transparency and Clarity of Mutual Expectations

- Describe in detail how to participate in class in respectful and inclusive ways.
  - Revisit these expectations before engaging in partner or group work.
- Have a conversation about mutual expectations. Share what you expect from students and provide space for students to offer what they expect from you.
- Use inclusive language to promote success for all students.
  - “I want everyone to be successful in learning in this course.”
  - “We will all engage in challenging tasks during this course, but this is a safe space for everyone to be pushed out of their comfort zone.”

Being transparent about what to expect and how to engage in your class decreases the cognitive load for students. Emphasizing that your course expectations are centered around respect and inclusivity sends the message that your classroom is a safe space. Additionally, the creation of mutual expectations disrupts the power imbalance that often exists between students and instructors and makes you more approachable.

## Student Feedback

- Allow students to provide anonymous feedback on their experience in your classroom at various points throughout the semester.
- See what trends exist in their responses and acknowledge them in your teaching practice throughout the remainder of the semester.
  - It may be helpful to share overall trends with the class and explicitly state what you will commit to starting, stopping, or continuing as a result of their feedback.

Allowing and listening to student voices builds trust. Students will be more likely to engage in classroom activities (which will increase learning outcomes) when they know that you are invested in fostering a safe space for them to learn.

# Level 2 Strategies

## Connection to Prosocial Topics

- Provide space in the curriculum to incorporate prosocial topics.
  - Prosocial topics could include: Public Health Issues, Solving Homelessness, Immigration Reform, Dispelling Violence, Food Deserts, Education Reform, etc.
- Create activities for students to investigate a subtopic within a prosocial topic and relay the information to their peers.
  - Relaying information could include: recording a video, creating a pamphlet, presenting a slideshow, poster session, small group facilitation, etc.

Engaging in rigorous material and connecting the material to prosocial topics will increase feelings of relevancy for students. By creating space for students to investigate their own topic of choice or providing a list of ideas, students are given the agency to intertwine their own identity with the course content. Student engagement will increase as they explore topics of interest and will provide space for students to learn from each other.

## Speed Intros

- Allow students to fill out a brief questionnaire (e.g., work habits, group work horror stories, communication preference, self-described personality).
- Students will pair up and exchange their questionnaires either verbally or by physically passing the paper.
- After three minutes, have the students rotate and introduce themselves to the next student.
- By the end of class, everyone should have introduced themselves.
- Have each student submit a list of the top three individuals they would feel comfortable working with and their questionnaire.
- Use the rankings and questionnaires to inform who is in each group for the rest of the semester.

Many students expressed fear of working with someone who is unaccepting of their identity and dislike of being paired with people who they don’t know. By structuring a space where students are able to quickly meet each other, answer questions about teamwork compatibility, and select their top three prospects for collaboration, students hold significant power in creating safe working groups for themselves.

## Student Generated Class Playlist

- Invite your students to share songs that resonate with them or reflect their identities, cultures, and interests.
  - Consider creating a digital form for them to share their song suggestions (with an option to complete the form anonymously).
- You can also add a few songs that you like to the playlist!
  - Start your class by playing music from the community generated playlist. You may even play music during lab or group work if students are not distracted by it.

Music is a great way to represent and learn more about the diverse backgrounds and identities in your classroom and invite students to be their full selves. As songs are playing, you and your students may talk about what the songs represent for you. This strategy will build community and create a more welcoming atmosphere for all students.
